# TUMOSPEC: A Nation-Wide Study of Hereditary Breast and Ovarian Cancer Families with a Predicted Pathogenic Variant Identified through Multigene Panel Testing

**DOI:** 10.3390/cancers13153659

**Published:** 2021-07-21

**Authors:** Fabienne Lesueur, Séverine Eon-Marchais, Sarah Bonnet-Boissinot, Juana Beauvallet, Marie-Gabrielle Dondon, Lisa Golmard, Etienne Rouleau, Céline Garrec, Mathilde Martinez, Christine Toulas, Tan Dat Nguyen, Fanny Brayotel, Louise Crivelli, Christine M. Maugard, Virginie Bubien, Nicolas Sevenet, Paul Gesta, Stéphanie Chieze-Valero, Sophie Nambot, Vincent Goussot, Véronique Mari, Cornel Popovici, Fabienne Prieur, Marie-Emmanuelle Morin-Meschin, Julie Tinat, Alain Lortholary, Hélène Dreyfus, Marie Bidart, Marie-Agnès Collonge-Rame, Monique Mozelle-Nivoix, Laurence Gladieff, Sophie Giraud, Nadia Boutry-Kryza, Jean Chiesa, Philippe Denizeau, Yves-Jean Bignon, Nancy Uhrhammer, Odile Cohen-Haguenauer, Paul Vilquin, Audrey Mailliez, Isabelle Coupier, Jean-Marc Rey, Elodie Lacaze, Odile Béra, Chrystelle Colas, Florence Coulet, Capucine Delnatte, Claude Houdayer, Christine Lasset, Jérôme Lemonnier, Michel Longy, Catherine Noguès, Dominique Stoppa-Lyonnet, Dominique Vaur, Nadine Andrieu, Olivier Caron

**Affiliations:** 1 Inserm, U900, Institut Curie, PSL Research University, Mines ParisTech, F-75005 Paris, France; Severine.Eon-Marchais@curie.fr (S.E.-M.); Sarah.Bonnet-Boissinot@curie.fr (S.B.-B.); juana.beauvallet@curie.fr (J.B.); marie-gabrielle.dondon@curie.fr (M.-G.D.); nadine.andrieu@curie.fr (N.A.); 2 Service de Génétique, Institut Curie, F-75005 Paris, France; lisa.golmard@curie.fr (L.G.); chrystelle.colas@curie.fr (C.C.); Dominique.StoppaLyonnet@curie.fr (D.S.-L.); 3 Service de Génétique, Département de Biologie et Pathologie Médicales, Gustave Roussy, F-94805 Villejuif, France; ETIENNE.ROULEAU@gustaveroussy.fr; 4 Laboratoire Génétique Moléculaire, Institut de Biologie, CHU Hôtel Dieu, F-44093 Nantes, France; celine.garrec@chu-nantes.fr; 5 Unité de Génétique Clinique, CHU Rouen Normandie, F-76100 Rouen, France; m.martinez@clinique-pasteur.com; 6 Laboratoire d’Oncogénétique, Institut Claudius Regaud, IUCT-Oncopole, F-31100 Toulouse, France; toulas.christine@iuct-oncopole.fr; 7 Institut de Cancérologie Jean Godinot, F-51100 Reims, France; TanDat.NGUYEN@reims.unicancer.fr (T.D.N.); fanny.brayotel@reims.unicancer.fr (F.B.); 8 Service d’Oncogénétique, Centre Eugène Marquis, F-35000 Rennes, France; l.crivelli@rennes.unicancer.fr; 9 Centre Paul Strauss, Unité d’Oncogénétique, ICANS Institut de Cancérologie de Strasbourg, F-67200 Strasbourg, France; christine.maugard@chru-strasbourg.fr; 10 Unité d’Oncogénétique, Département de Bio-Pathologie, Institut Bergonié, F-33000 Bordeaux, France; v.bubien@bordeaux.unicancer.fr; 11 Institut Bergonié, UFR des Sciences Pharmaceutiques, Collège des Sciences de la Santé Université de Bordeaux, INSERM U1218, F-33000 Bordeaux, France; n.sevenet@bordeaux.unicancer.fr; 12 CH Georges Renon, Service d’Oncogénétique Régional Poitou-Charentes, F-79000 Niort, France; paul.gesta@ch-niort.fr (P.G.); stephanie.chieze-valero@ch-niort.fr (S.C.-V.); 13 Institut GIMI, CHU de Dijon, Hôpital d’Enfants, F-21000 Dijon, France; sophie.nambot@chu-dijon.fr; 14 Unité de Biologie Moléculaire, Département de Biologie et Pathologie des Tumeurs, Centre Georges François Leclerc, F-21000 Dijon, France; vgoussot@cgfl.fr; 15 Service d’Oncogénétique et Recherche Clinique, Centre Antoine Lacassagne, F-06100 Nice, France; veronique.mari@nice.unicancer.fr; 16 Département d’Anticipation et de Suivi des Cancers, Oncogénétique Clinique, Institut Paoli-Calmettes, F-13009 Marseille, France; popovicic@ipc.unicancer.fr (C.P.); NOGUESC@ipc.unicancer.fr (C.N.); 17 Service de Génétique, CHU—Hôpital Nord, F-42055 Saint Etienne, France; fabienne.prieur@chu-st-etienne.fr; 18 Service d’Oncogénétique, Institut de Cancérologie de l’Ouest, Site Paul Papin, F-49055 Angers, France; marie-emmanuelle.morin-meschin@ico.unicancer.fr; 19 Génétique Médicale, CHU de Bordeaux, F-33000 Bordeaux, France; julie.tinat@chu-bordeaux.fr; 20 Service d’Oncologie Médicale, Centre Catherine de Sienne, Hôpital Privé du Confluent, F-44200 Nantes, France; alain.lortholary@groupeconfluent.fr; 21 Clinique Sainte Catherine, F-84000 Avignon, France; h.dreyfus@isc84.org; 22 Département de Génétique, Hôpital Couple-Enfant, CHU de Grenoble, F-38700 Grenoble, France; 23 Laboratoire de Génétique Moléculaire: Maladies Rares et Oncologies, Institut de Biologie et Pathologie, CHU Grenoble Alpes, F-38700 Grenoble, France; mbidart@chu-grenoble.fr; 24 Consultation d’Oncogénétique, Oncobiologie Génétique Bioinformatique, CHRU, F-25000 Besançon, France; ma1collongerame@chu-besancon.fr; 25 Centre Hospitalier de Troyes, Hôpital S. Veil, F-10000 Troyes, France; Monique.Mozelle@ch-troyes.fr; 26 Service d’Oncologie Médicale, Institut Claudius Regaud—IUCT-Oncopole, F-31100 Toulouse, France; Gladieff.Laurence@iuct-oncopole.fr; 27 Hospices Civils de Lyon, Service de Génétique, Groupement Hospitalier EST, F-69500 Bron, France; sophie.giraud@chu-lyon.fr (S.G.); nadia.boutry-kryza@lyon.unicancer.fr (N.B.-K.); 28 UF de Cytogénétique et Génétique Médicale, CHRU Hôpital Caremeau, F-30900 Nîmes, France; jean.chiesa@chu-nimes.fr; 29 Service de Génétique Clinique, Hôpital Sud, F-35200 Rennes, France; philippe.denizeau@chu-rennes.fr; 30 Centre Jean Perrin, Département d’Oncogénétique, Université Clermont Auvergne, UMR INSERM 1240, F-63011 Clermont Ferrand, France; Yves-Jean.BIGNON@clermont.unicancer.fr; 31 Centre Jean Perrin, LBM OncoGenAuvergne, F-63011 Clermont Ferrand, France; nancy.uhrhammer@clermont.unicancer.fr; 32 Unité d’Oncogénétique Clinique AP-HP Nord-UP, DMU Icare, UFR de Médecine de l’Université de Paris, INSERM UMR-S 976, Hôpital Saint-Louis, F-75010 Paris, France; odile.cohen-haguenauer@aphp.fr; 33 Département de Génomique des Tumeurs Solides, Hôpital Saint-Louis, APHP, F-75010 Paris, France; paul.vilquin@aphp.fr; 34 Centre Oscar Lambret, F-59000 Lille, France; a-mailliez@o-lambret.fr; 35 Service de Génétique Médicale et Oncogénétique, Hôpital Arnaud de Villeneuve, CHU Montpellier, F-34090 Montpellier, France; i-coupier@chu-montpellier.fr; 36 INSERM 896, CRCM Val d’Aurelle, F-34090 Montpellier, France; 37 Laboratoire de Biopathologie Cellulaire et Tissulaire des Tumeurs, CHRU Arnaud de Villeneuve, F-34090 Montpellier, France; jm-rey@chu-montpellier.fr; 38 Unité de Génétique Médicale, Groupe Hospitalier du Havre, F-76290 Le Havre, France; elodie.lacaze@ch-havre.fr; 39 CHU de Martinique, F-97261 Fort-de-France, France; odile.bera@chu-martinique.fr; 40 Service de Génétique, AP-HP, Hôpital Universitaire Pitié-Salpétrière, F-75013 Paris, France; florence.coulet@aphp.fr; 41 Unité d’Oncogénétique, ICO-Site René Gauducheau, F-44800 Nantes Saint Herblain, France; Capucine.Delnatte@ico.unicancer.fr; 42 Département de Génétique, Hôpital Universitaire de Rouen, Unirouen, Inserm U1245, F-76000 Rouen, France; Claude.Houdayer@chu-rouen.fr; 43 CNRS UMR 5558, Université Claude Bernard Lyon 1, F-69100 Villeurbanne, France; christine.lasset@lyon.unicancer.fr; 44 Centre Léon Bérard, Unité de Prévention et Epidémiologie Génétique, F-69008 Lyon, France; 45 R&D UNICANCER, Fédération Nationale des Centres de Lutte Contre le Cancer, F-75013 Paris, France; j-lemonnier@unicancer.fr; 46 Cancer Genetics Unit & INSERM U1218, Institut Bergonié, University of Bordeaux, F-33000 Bordeaux, France; M.Longy@bordeaux.unicancer.fr; 47 Institut Paoli-Calmettes & Aix Marseille University, INSERM, IRD, SESSTIM, F-13009 Marseille, France; 48 Inserm, U830, Université de Paris, F-75005 Paris, France; 49 Laboratoire de Biologie et de Génétique du Cancer, Centre François Baclesse, F-14000 Caen, France; D.VAUR@baclesse.unicancer.fr; 50 Département de Médecine Oncologique, Gustave Roussy, F-94805 Villejuif, France; 51 Centre Médical de Bligny, F-91640 Briis-Sous-Forges, France

**Keywords:** genetic predisposition to breast and ovarian cancer, cancer risk estimate, multigene panels, penetrance of pathogenic variants

## Abstract

**Simple Summary:**

TUMOSPEC was designed for estimating the risk of cancer for carriers of a predicted pathogenic variant (PPV) in a gene usually tested in a hereditary breast and ovarian cancer context. Index cases are enrolled consecutively among patients who undergo genetic testing as part of their care plan in France. First- and second-degree relatives and cousins of PPV carriers are invited to participate whether they are affected by cancer or not, and are tested for the familial PPV. Genetic, clinical, family and epidemiological data are centralized at the coordinating centre. The three-year feasibility study included 4431 prospective index cases, with 19.1% of them carrying a PPV. This showed that the study logistics are well adapted to clinical and laboratory constraints, and collaboration between partners (clinicians, biologists, coordinating centre and participants) is smooth. Hence, TUMOSPEC is being pursued, with the aim of optimizing clinical management guidelines specific to each gene.

**Abstract:**

Assessment of age-dependent cancer risk for carriers of a predicted pathogenic variant (PPV) is often hampered by biases in data collection, with a frequent under-representation of cancer-free PPV carriers. TUMOSPEC was designed to estimate the cumulative risk of cancer for carriers of a PPV in a gene that is usually tested in a hereditary breast and ovarian cancer context. Index cases are enrolled consecutively among patients who undergo genetic testing as part of their care plan in France. First- and second-degree relatives and cousins of PPV carriers are invited to participate whether they are affected by cancer or not, and genotyped for the familial PPV. Clinical, family and epidemiological data are collected, and all data including sequencing data are centralized at the coordinating centre. The three-year feasibility study included 4431 prospective index cases, with 19.1% of them carrying a PPV. When invited by the coordinating centre, 65.3% of the relatives of index cases (5.7 relatives per family, on average) accepted the invitation to participate. The study logistics were well adapted to clinical and laboratory constraints, and collaboration between partners (clinicians, biologists, coordinating centre and participants) was smooth. Hence, TUMOSPEC is being pursued, with the aim of optimizing clinical management guidelines specific to each gene.

## 1. Introduction

DNA-based testing has become a common part of routine clinical assessment for individuals with clinical features suggestive of a hereditary predisposition. For hereditary breast and ovarian cancer (HBOC) predisposition, clinical genetic testing has focused primarily on the two major predisposing genes: *BRCA1* and *BRCA2 (BRCA1/2).* The identification of a germline disease-causing variant, also called “pathogenic variant”, in the index case of an HBOC family (i.e., the first ascertained patient) allows her/his relatives to benefit from predictive testing and to receive genetic counselling and preventive medical management [1]. Owing to improvements in knowledge about carcinogenesis pathways and to the advent of sequencing technologies, other DNA repair genes have been confirmed or have more recently emerged as HBOC susceptibility genes, such as *ATM*, *CHEK2*, *PALB2*, *RAD51C* and *RAD51D* [2,3,4]. Massive parallel sequencing has also deeply changed the clinical approach to genetic testing in medical oncology. Instead of single gene testing, it provides clinicians with information about one or more germline pathogenic variants associated with disorders/syndromes in a single test. In 2018, 21,217 subjects attended one of the 149 cancer genetics clinics in France for advice about their personal and/or family history of breast and/or ovarian cancer. Among them, 18,633 index cases had a multigene panel test, and a pathogenic variant was identified in 10% of them [5]. 

While clinical geneticists agree to define a gene analysis as usable only if the identification of a pathogenic variant results in a health benefit for patients, very few studies have investigated the clinical validity of the inclusion of multicancer syndrome genes and of the other breast and ovarian cancer susceptibility genes in multigene panels used in the context of clinical management of HBOC family members [6]. To date, only the risks of breast and ovarian cancer for carriers of a loss-of-function (LoF) variant in *PALB2* [7,8], *RAD51C* and *RAD51D* [9] have been assessed by collecting data on variant carriers and their relatives from multiple centres worldwide to reach satisfactory statistical power. The published data suggest that the breast cancer risk for *PALB2* LoF carriers may overlap with that of *BRCA2* pathogenic variant carriers [8]. In contrast, *RAD51C* and *RAD51D* LoF confer a moderate risk of breast cancer but a high enough risk of tubo-ovarian carcinoma, which leads to the recommendation of a risk-reducing salpingo-oophorectomy as a preventative measure in these women [9]. However, even for these three genes, gathering more family data will help to refine the estimate of cancer risk for variant carriers. For other genes included in commercial or custom in-house HBOC multigene panels, the reliability of associated age-dependent cancer risks, and the clinical utility of testing them have not been demonstrated. Moreover, genotype–phenotype correlations, as well as other potential modifying factors, need to be investigated.

Another limitation for the use of all these genes in clinical practice is the unknown pathogenicity of many identified variants for a given disease [10,11]. Indeed, it is challenging to classify many of them as either “pathogenic”, “likely pathogenic”, “of uncertain clinical significance”, “likely benign” or “benign”. A survey conducted on 16 genes commonly included in HBOC panels (*ATM*, *BARD1*, *BRIP1*, *CDH1*, *CHEK2*, *MRE11A*, *NBN*, *NF1*, *PALB2*, *PTEN*, *RAD50*, *RAD51C*, *RAD51D*, *STK11*, *TP53* and *MEN1*) among members of the Evidenced-based Network for the Interpretation of Germline Mutant Allele (ENIGMA) [12] confirmed that currently only a small number of genes beyond *BRCA1*/*2* are routinely analyzed worldwide. For those, only the variants defined as “pathogenic”, i.e., essentially LoF, are used in clinics [13]. Management guidelines for carriers are very conservative and the identification of such a variant does not greatly impact the usual practices based on family history. Moreover, these guidelines differ between countries, especially in regard to starting age and type of imaging, and risk-reducing surgery recommendations [13]. 

TUMOSPEC (for “TUMOr SPECtrum”) is a family-based nation-wide study designed to measure the age-dependent cancer risk of carriers of a predicted pathogenic variant (PPV) in a gene usually included in diagnostic HBOC multigene panels. It also aims at defining the tumor spectrum associated with these genes, i.e., the variety of organs concerned by the predisposition, in order to provide consensual clinical recommendations specific to each gene. The study is conducted in partnership with the French network of family cancer clinics and molecular diagnostics laboratories that compose the Cancer and Genetic Group (http://www.unicancer.fr/en/cancer-and-genetic-group, accessed on 19 July 2021). The TUMOSPEC multigene panel includes “actionable” genes other than *BRCA1* and *BRCA2* (i.e., genes that are nowadays routinely tested in France in addition to the two major predisposing genes if an HBOC predisposition is suspected) and “research” genes (i.e., genes for which no consensus clinical management guidelines have been proposed thus far in France [14]). Index cases are enrolled consecutively among patients who are being offered a genetic test as part of their care plan. When an actionable variant is detected, the counselled family members are invited by the clinical geneticist to participate in the study; otherwise, family members of PPV carriers are invited by the coordinating centre that centralizes genetic, clinical, family and epidemiological data for all participants. Here, we describe the study protocol and results of the three-year feasibility study, which included 4431 prospective index cases. For families where an actionable variant was detected in the index case, 1.8 counselled relatives per family, on average, were enrolled by the clinical geneticist through the usual cascade testing. For other families where a non-actionable variant was detected, 5.7 relatives per family, on average, were enrolled by the coordinating centre. Regardless of the type of gene or the mode of invitation of relatives, we found that the proposed logistics were well adapted to clinical and laboratory constraints, and that communication between partners (clinics, laboratories, coordinating centre and participants) (The clinics and diagnostics laboratories composing the TUMOSPEC Investigators Group are shown in Appendix A) was quite smooth. Therefore, TUMOSPEC is being pursued to assess cancer risks in the families of PPV carriers. In future, the TUMOSPEC protocol may be easily adapted to other hereditary cancers or other diseases.

## 2. Materials and Methods

### 2.1. Family Enrolment

#### 2.1.1. Index Case Definition and Eligibility of Family Members

An index case is the first member of a family being counselled who has never undergone any genetic testing in the past and who has been recommended for an HBOC multigene panel test, which includes *BRCA1* and *BRCA2* testing, as part of her/his care plan at enrolment in TUMOSPEC. First- and second-degree relatives and cousins from both sides of the family are eligible for the study if a variant identified in the index case fulfils the variant eligibility criteria (see Section 2.2). Index cases and relatives should be aged 18 years or older. Children of index cases, even older than 18 years, are not eligible for the study as these individuals will not be informative enough in the analyses. 

#### 2.1.2. Process for Invitation of Family Members Depends on the Altered Gene and on the Class of Variant

**Figure 1:**
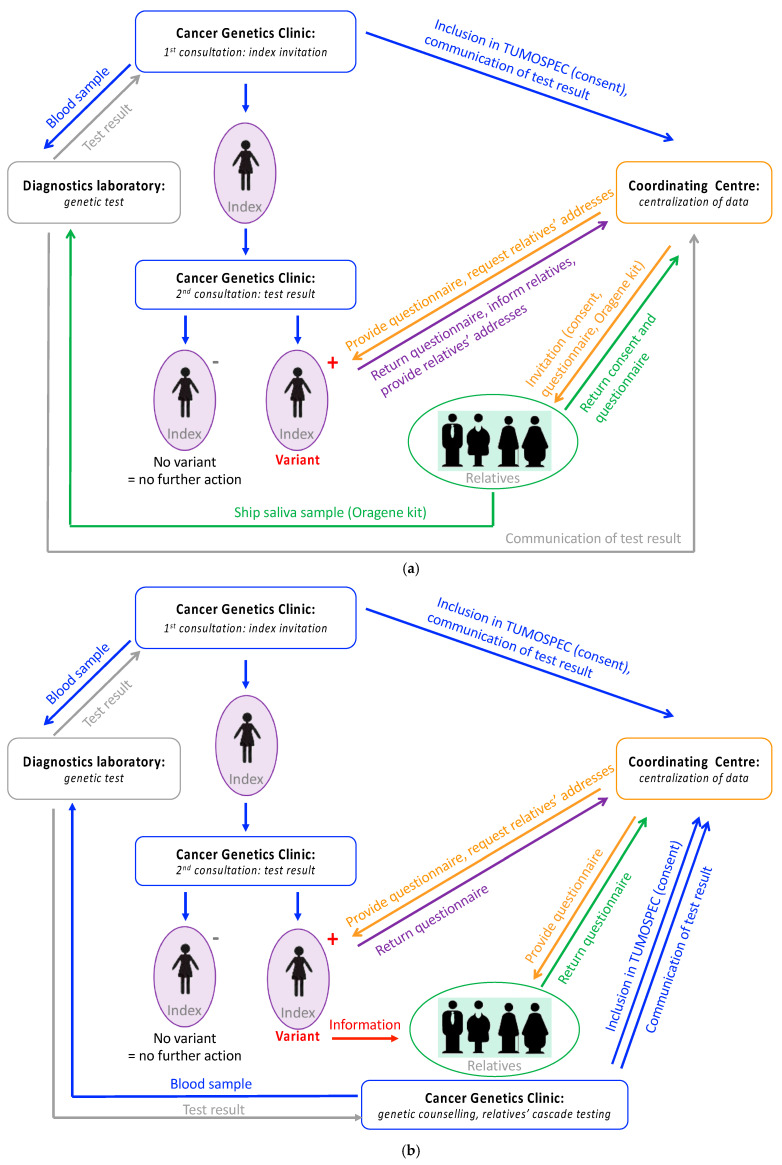
TUMOSPEC protocol. (**a**) Process for the collection of data in families segregating a variant in a gene from sub-panel A or a variant of unknown clinical significance in a gene from sub-panel B. (**b**) Process for the collection of data in families segregating a pathogenic variant in a gene from sub-panel B.

Genes included in the TUMOSPEC panel were selected by a steering committee composed of clinical practitioners, epidemiologists and molecular geneticists. A gene was selected if it had been linked with breast and/or ovarian cancer predisposition in several independent case–control or family-based studies, including studies on familial breast cancer conducted by investigators in the French population [15,16,17,18]. The TUMOSPEC multigene panel is divided into sub-panel A, which includes genes for which no consensus clinical management guidelines have been proposed thus far in France (namely *ATM*, *BAP1*, *BARD1*, *BRIP1*, *CHEK2*, *FAM175A*, *FANCM*, *MRE11A*, *NBN*, *RAD50*, *RAD51B*, *RINT1*, *STK11* and *XRCC2*) and sub-panel B, which includes genes for which consensus clinical management guidelines exist for pathogenic variant carriers (namely *CDH1*, *PALB2*, *MLH1*, *MSH2*, *MSH6*, *PMS2*, *PTEN*, *RAD51C*, *RAD51D* and *TP53*) [14]. Therefore, the prediction on the pathogenicity of the identified variant determines how relatives of index cases are invited to participate to the study. The two protocol options are summarized in Figure 1a (sub-panel A) and 1b (sub-panel B). 

If a variant eligible for TUMOSPEC is found in a gene from sub-panel A, the name of the gene is not revealed to the index case since it will not modify her/his management or the management of her/his relatives. In this situation, the coordinating centre invites directly eligible relatives (i.e., first- and second-degree relatives, and cousins) to participate in the study. If an eligible variant is detected in a gene from sub-panel B and classified as “pathogenic” or “Class 5”, according to the five-tier class system defined by Plon et al. [11], the name of the gene is revealed to the index case since it will modify her/his management and the management of her/his relatives. In that case, the geneticist invites the family members to participate in the study during a genetic counselling session. If a variant in a gene from sub-panel B is classified other than “pathogenic” or “Class 5”, the process for inviting the relatives of the index case is as for variants detected in genes from sub-panel A.

In addition to the index cases included prospectively in the study, some individuals carrying a variant identified through multigene panel testing prior to the recruitment of the prospective cases were also invited to participate; this was to assess more rapidly the feasibility of including relatives and to validate our logistics. They are hereafter referred to as “retrospective index cases”.

### 2.2. Variants Eligibility Criteria

A variant identified in one of the TUMOSPEC genes is eligible for the study, and therefore considered as a PPV, if it fulfils the following criteria:

- The minor allele frequency (MAF) is less than 0.05% in the general population in all seven ethnic groups: non-Finnish European (EUR), Finnish (FIN), East Asian (EA), South Asian (SA), Latino (LAT), African (AFR) and Ashkenazi Jewish (AJ) according to the 1000Genomes [19] and GnomAD [20] databases. Of note, an exception was made for two variants with MAF > 0.05% (namely NM_007194.4:c.1100del (p.Thr367fs; rs555607708) in *CHEK2* [21,22] and NM_020937.4:c.5791C>T (p.Arg1931*; rs144567652) in *FANCM* [23,24,25]) during the course of the feasibility study (i.e., since October 2019) because of their already reported association with breast cancer risk in previous studies.
- The effect of the variant is predicted to have a deleterious impact on the gene product function. More specifically, eligible variants are:
  - Variant predicted to shorten the coding sequence of the gene (nonsense variants, small insertions/deletions (indels), canonical splice site alterations and large rearrangements leading to a truncated protein). Such variants are also referred to as “loss-of-function variants” or “LoF”;
  - Genetic alterations in which a single base pair substitution alters the genetic code, referred to as “missense variants” and in-frame indels (small insertions/deletions that do not alter the reading frame) if:
    - They have been classified as “pathogenic” or “Class 5” according to the five-tier class system defined by Plon et al. [11], by a group of experts for a specific gene (typically ClinVar or ENIGMA classification expert groups);
    - Or an in vitro assay has demonstrated the deleterious impact on the gene product function or on splicing;
    - Or the score obtained with the Combined Annotation Dependent Depletion (CADD) tool [26] is indicative of the deleteriousness of the variant. Here, we considered variants with a CADD phred score ≥20 eligible for the study. A score of 20 means that the variant is among the top 1% of most deleterious substitutions when ranking all possible substitutions in the human genome. For genes for which a manually curated protein multiple sequence alignment is available on the Align-GVGD website (http://agvgd.hci.utah.edu/agvgd_input.php, accessed on 1 July 2020), namely *ATM*, *CHEK2*, *MLH1*, *MRE11A*, *MSH2*, *MSH6*, *NBN*, *PALB2*, *PMS2*, *RAD50*, *TP53* and *XRCC2*, missense variants with Align-GVGD grade C45, C55 or C65 are also eligible [27], even if the CADD phred score is <20.

All identified variants are subject to a curation process and named according to the Human Genome Variant Society (HGVS) nomenclature before integration in the TUMOSPEC genetic database. Although *BRCA1* and *BRCA2* are not part of the TUMOSPEC panel, the two genes are HBOC genes and they are therefore tested in parallel with the 24 investigated genes here. The co-occurrence of multiple eligible variants in one or more of the TUMOSPEC genes, or with a *BRCA1/2* variant (either a pathogenic variant or a variant of uncertain clinical significance (VUS)), is recorded in the database. 

### 2.3. Biological Samples and Genetic Analyses

For index cases, the TUMOSPEC panel is analysed on the DNA aliquot prepared from the same EDTA blood sample that is used to perform the routine HBOC multigene panel test; the genetic analysis is performed by the laboratory performing the *BRCA1/2* test. A second blood sample, usually a sample stored on an FTA^®^ card, is collected to confirm the presence of the variant following routine practice. 

The participating laboratories analyse the full coding sequence and exon–intron boundaries of at least 15 out of 24 genes in the TUMOSPEC panel using their usual (or upgraded) hybridization capture kit and sequencing instrument (Table S1). Some heterogeneity may be introduced due to a difference in the in-house bioinformatics pipelines implemented in each laboratory. However, the standard quality procedures used for detecting variants in actionable genes are applied to all genes, and all eligible variants identified in index cases are confirmed by Sanger sequencing (for single nucleotide variants or small indels), Multiplex Ligation-dependent Probe Amplification (MLPA) [28] or Quantitative Multiplex Polymerase chain reaction of Short fluorescent Fragments (QMPSF) [29] (for large indels and rearrangements).

Relatives invited by the coordinating centre provide a saliva sample using an Oragene^®^ DNA sample kit (OG-500.014) and send it to the laboratory that has tested the index case. Pre-stamped envelopes are provided to the relatives so that the samples can be sent to the appropriate laboratory by postal mail; temperature-controlled conditions are not required. DNA is extracted, analysed and stored applying the standard procedures that are used for all diagnostic tests. The relative’s genotype for the variant detected in the index case is determined by Sanger sequencing (for single nucleotide variants or small indels), MLPA [28] or QMPSF [29] (for large indels and rearrangements).

Blood and saliva samples, as well as DNA aliquots from all participants, are kept in the laboratory after the genetic analysis has been performed, for future research projects that will be conducted in the framework of TUMOSPEC. No systematic collection of tumor specimens is performed. However, pathology reports and information of sample storage conditions and location are collected. This information will facilitate access to the tumor samples for specific projects to come.

### 2.4. Data Collection and Storage

All index cases carrying an eligible variant and all relatives affected and unaffected with cancer participating in the research (whether they carry the familial variant or not) complete a questionnaire on environmental, lifestyle and personal and family history of cancer (and other diseases). This self-report questionnaire contains questions about demographics, lifestyle (alcohol intake, smoking, etc.) and medical radiation exposures, as well as gynecological and obstetric history for women. Questionnaires are collected by the coordinating centre, where data are coded, digitized and checked for inconsistencies. Requests for additional information are sent out to the participants in case some information is missing or incoherent.

A database gathers familial, clinical and epidemiological data for each participant, and another database centralizes the results of the genetic analyses performed by the laboratories. All the data are stored on secure servers in a manner guaranteeing patient anonymity. Only the staff of the coordinating centre and study have access to the epidemiological and genetic data.

For index cases, next-generation sequencing (NGS) data (FASTQ format) are also centralized by the coordinating centre for future downstream analyses. This will allow, for example, the comparison of the performance of the different routine bioinformatics pipelines in terms of NGS read quality control, NGS read alignment and reference mapping, variant frequency measurements, analytical sensitivity and specificity, and variant annotation tools in order to harmonize the reporting of the variants.

## 3. Results

### 3.1. Data Collected

**Figure 2:**
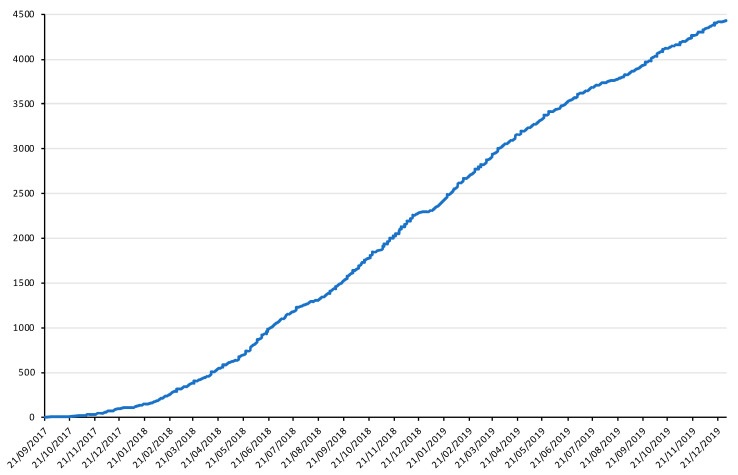
Dynamics of recruitment of prospective index cases during the feasibility study (September 2017–December 2019). X axis: calendar time scale; Y axis: cumulative number of index cases enrolled in the study.

A total of 37 family cancer clinics and 16 molecular diagnostics laboratories participated in the feasibility study (Tables S1 and S2). The recruitment of index cases started in September 2017 and ended in December 2019, and the recruitment of relatives ended in July 2020. In total, 4431 prospective and 71 retrospective index cases were recruited. Figure 2 shows the dynamics of recruitment of the prospective index cases during the feasibility study. The description of available data per type of participants is presented in Table 1. Among the 2389 prospective index cases with a genetic result available by December 2019, 456 (19.1%) individuals carried at least one PPV matching the TUMOSPEC variant eligibility criteria (see Methods). A total of 426 relatives belonging to 48 families were invited by the coordinating centre, and 278 (65.3%) of them had consented to participate by July 2020. The average number of participating relatives per family was therefore 5.7 (range: 0–19). During the same period of time, 28 counselled relatives from 16 families segregating an actionable variant were recruited by a clinical geneticist, i.e., 1.8 relatives per family on average (range: 1–4).

The epidemiology questionnaire was sent to 812 participants (417 index cases and 246 relatives from the prospective dataset, and 71 index cases and 78 relatives from the retrospective dataset). By July 2020, 193 (39.5%) index cases and 200 (61.7%) relatives had completed and returned their questionnaire (Table 1). The difference in return rates between index cases and relatives (Figure S1) may be explained by the fact that the index cases sent back their questionnaire to the coordinating centre together with a list of their relatives who were eligible for the study. Before doing so, the index cases first contacted their relatives to inform them about the study and protocol, and requested their permission to provide their mail addresses and other contact details to the investigators, which meant a much longer delay in returning the questionnaire for the index cases than for the relatives.

Relatives invited by the coordinating centre received the epidemiological questionnaire along with an Oragene kit for saliva collection. Fifty percent of relatives who returned the questionnaire to the coordinating centre did so in less than 27 days (range: 5–397), and the delay in sending their saliva sample to the diagnostic laboratory was similar (median: 24 days, range 2–384). Of note, the delay in sending the saliva sample to the laboratory was calculated after excluding individuals who sent it after 16 March 2020, which corresponds to the start of the first national lockdown in France due to the COVID-19 pandemic (as the collection of saliva samples during this period was interrupted).

### 3.2. Participants’ Characteristics

Among the 4502 participating index cases, 4419 were women and 83 were men. Mean age at recruitment was 52.5 years (range: 19–91) for women and 65.1 years (range: 35–90) for men. Ninety-three percent of index cases had developed a first cancer prior to recruitment. Among female index cases, 3779 (85.5%) had breast cancer, 462 (10.5%) had ovarian cancer, 129 (2.9%) had cancer at another site, and 49 (1.1%) had no cancer. Among male index cases, 60 (72.2%) had breast cancer, 14 (17.0%) had prostate cancer, 8 (9.6%) had cancer at another site and 1 had no cancer (1.2%). For female index cases, we did not observe any difference in the mean age at diagnosis of first cancer according to the result of the TUMOSPEC panel analysis (46.9 years for carriers of a PPV vs. 47.4 years for noncarriers), nor between prospective (46.9 years) and retrospective cases (47.7 years) (Table 2). In the prospective dataset, male index cases carrying a PPV were diagnosed at a younger age than noncarriers (54.4 years vs. 61.9 years). Male index cases in the retrospective dataset, who by design are PPV carriers, were even younger at cancer diagnosis (mean: 48.5 years), which may be attributable to a selection bias (Table 2).

Out of 386 participating relatives, 60 (15.5%) had had cancer at enrolment in the study. The mean age at diagnosis of first cancer was 53.3 years (range: 21–83) for women and 63.9 years (range: 33–87) for men. Out of 43 affected female relatives, 31 (72.1%) had breast cancer, and 10 out of 15 affected male relatives (66.6%) had prostate cancer. Other first cancers for women were cervical cancer (*N* = 4), thyroid cancer (*N* = 3), melanoma (*N* = 2), ovarian cancer (*N* = 1), endometrial cancer (*N* = 1) and colon cancer (*N* = 1). Other first cancers for men were lung cancer (*N* = 2), lymphoma (*N* = 2) and testicular cancer (*N* = 1).

### 3.3. Identified Variants

**Figure 3:**
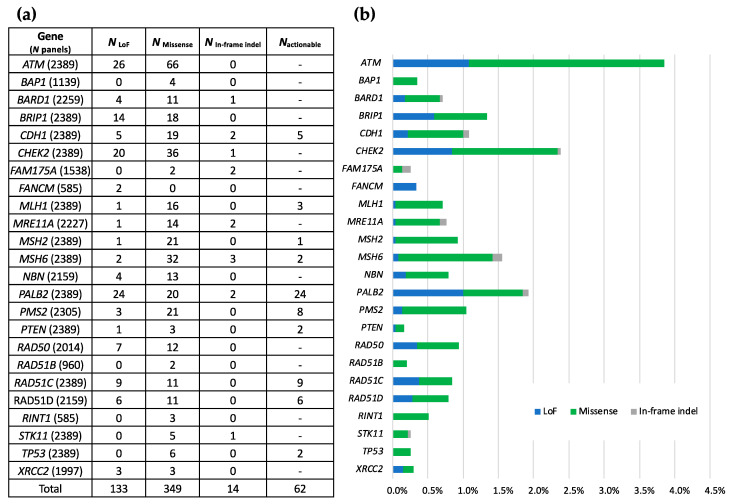
Distribution of eligible variants identified in the prospective index cases, per gene and per variant type. (**a**) Number of loss of function variants (*N*_LoF_), missense variants (*N*_missense_), in-frame indels (*N*_indel_) and actionable variant (*N*_actionable_) per gene. *N*_panels_: number of analysed panels containing the gene. (**b**) Weighted distribution of variants per gene (number of detected variants/*N*_panels_).

In total, 133 LoF (26.8%), 349 missense variants (70.4%) and 14 in-frame indels (2.8%) were detected among the 456 prospective index cases with positive TUMOSPEC panel results (Figure 3a). A total of 40 index cases carried 2 eligible variants and no index cases carried 3 or more variants. A total of 62 index cases carried a variant classified as pathogenic in an actionable gene (sub-panel B) and 154 index cases carried a VUS in this group of genes, that is a Class 3 variant according to the 5-tier classification [11] (data not shown). The weighted distribution of variants per gene is shown on Figure 3b. The most frequently altered genes in the TUMOSPEC panel were *ATM*, *CHEK2*, *PALB2*, *MSH6* and *BRIP1*. 

Additionally, 82 eligible variants were detected in the 71 retrospective index cases (53 LoF, 28 missense and 1 indel). In this series, 41 variants were in an actionable gene, of which 26 were classified as pathogenic and 15 were classified as VUS. However, the distribution of variants in the retrospective series does not reflect the true distribution of variants in index cases of HBOC families, given that some genes were not part of the HBOC panels used by the laboratories prior to the implementation of the TUMOSPEC protocol, and some variant types were not flagged by the analytical pipelines implemented in the laboratories for routine genetic testing. Moreover, clinicians may have selected retrospective cases on the family phenotype or the deleteriousness of the variant. 

Although *BRCA1* and *BRCA2* were not, per se, part of the TUMOSPEC panel, the two genes were tested in parallel with the investigated genes, which allowed us to assess the co-occurrence of *BRCA1/2* pathogenic variants with TUMOSPEC eligible variants in index cases. We found that 32 out of the 456 (7.0%) prospective index cases carrying at least 1 eligible variant also carried a *BRCA1/2* pathogenic variant **(**Table 2). As expected, the frequency of *BRCA1/2* pathogenic variants in the retrospective index cases was much lower than the one observed in the prospective series, as retrospective index cases with no *BRCA1/2* variants were more likely to have been invited to participate in TUMOSPEC in an attempt to elucidate the familial predisposition (Table 2). 

The complete list of variants in the TUMOSPEC genes identified in the prospective and retrospective index cases of the feasibility study and their occurrence is provided in Table S3. It should be noted that the two variants NM_007194.4:c.1100del (p.Thr367fs; rs555607708) in *CHEK2* [21,22] and NM_020937.4:c.5791C>T (p.Arg1931*; rs144567652) in *FANCM* [23,24,25] were under-reported (0 carrier of the *FANCM* variant and only 8 carriers of the *CHEK2* variant). This is because their minor allele frequency exceeds 0.05% in populations of the 1000Genomes project [19] and in the Genome Aggregation Database (GnomAD) [20], and they were therefore initially not eligible for this study. However, due to their relevance in breast cancer susceptibility, an exception was made to include these two variants from October 2019.

## 4. Discussion

The identification of a genetic predisposition to cancer is now an integral part of the clinical care of patients and their relatives. It allows the implementation of prevention programs and screening when the risks are known. The effectiveness of genetic testing has been notably demonstrated for women carrying a *BRCA1* or *BRCA2* pathogenic variant and for whom prophylactic surgeries reduce mortality. However, current genetic testing does not provide significant assistance when no pathogenic variant is identified, that is, 85% of the cases of HBOC families enrolled in TUMOSPEC. New genetic tests are essential to support clinical decision making and to ensure improved outcomes in this situation. Current multigene panel tests often combine both diagnosis and research genes, but the genes sequenced for research purpose should be defined and patients informed before testing [30]. Note that the classification of a given gene as diagnosis or research might change in the coming years. In particular, *STK11* is not currently an actionable gene for HBOC in France, unlike in the USA or other countries following the National Comprehensive Cancer Network guidelines [31]. So far, no *STK11* pathogenic variants for Peutz-Jeghers syndrome have been identified in TUMOSPEC. However, should such variants be identified in the future and cascade testing be performed in the family, the invitation of family members should be handled by the clinical geneticist. Multigene panel sequencing will have the potential to improve germline risk assessment in HBOC families if: 1. classification of variants regarding their pathogenicity is accurate; 2. reliable estimates of the associated age-specific cancer risks can be obtained; and 3. a consensus is made on when to test for a given gene and how to manage a reported (likely) pathogenic variant [2,6]. However, for some genes, the cumulative cancer risk for carriers of a pathogenic variant may be found to be quite low, and testing such genes would not improve the surveillance of the patients. Conversely for other genes, carriers of a pathogenic variant may benefit from adapted surveillance and treatment. 

The TUMOSPEC feasibility study included 4431 prospective index cases, and 19.1% of the prospective index cases with an available genetic result were found to carry at least one PPV in a gene on the investigated panel. Furthermore, 65.3% of the relatives of PPV carriers who were directly invited by the coordinating centre agreed to participate, with 50% of them returning their questionnaire and saliva sample in less than 1 month. On average, 5.7 relatives per positive family invited by the coordinating centre agreed to participate (i.e., 278 relatives from 48 families), while only 1.8 relatives per family (i.e., 28 relatives from 16 families) were enrolled by the clinician who counselled the family members following the identification of an actionable variant in the index case. This shows the efficiency of having family members invited by a coordinating centre in such a research program. 

Qualitative feedback from clinicians and diagnostics laboratories teams regarding recruitment methods, information and sampling circuits, etc., as well as the communication between the coordinating centre, clinicians, laboratories and participants (particularly the efficiency of recruiting relatives), and the comprehension of documents (newsletter, consent forms, questionnaire, etc.) satisfied the evaluation criteria, therefore the study is being pursued. Overall, expanding the study to the analysis of 10,000 multigene panels within the next 3 years will identify ~2000 families with a PPV in one of the 24 genes, with two to six family members genotyped for the familial variant, which will allow the refinement of cancer risk estimates for the most frequently altered genes. 

The PPV rates according to gene and family phenotype will define our analysis strategy. For instance, we expect that we will rapidly gather sufficient families for genes such as *ATM*, *CHEK2*, *PALB2*, *MSH6* and *BRIP1*, while *PTEN*, *STK11*, *FANCM* and *XRCC2* families will be much rarer. For this latter group of genes, the data may be compiled with data from other countries where similar efforts have been initiated and/or in the framework of international consortia such as ENIGMA (https://enigmaconsortium.org/, accessed on 19 July 2021), BRIDGES (https://bridges-research.eu/project-bridges/, accessed on 19 July 2021) and COMPLEXO [32].

Our analytical strategy to assess cancer risks has been elaborated on the fact that the TUMOSPEC families are ascertained through family cancer clinics for the HBOC phenotype. Therefore, once we have recruited enough families who segregate a PPV for a given gene, we will use methods such as the genotype-restricted likelihood method and maximum likelihood parametric methods, which provide unbiased penetrance estimates irrespective of the criteria used for family selection [33] or other modified segregation-analysis approaches, such as MENDEL [34]. These methods use information available in families by calculating likelihood conditioned on the phenotypes of all family members (retrospective likelihood), also allowing for residual familial aggregation additional to the effect of PPVs. In order to capture the potential nature of the multiple cancers associated with some genes, we will also use competing risk models [35]. For some genes, the rarity of families who segregate an eligible variant may make the estimation of cumulative risks impossible in the TUMOSPEC sample (e.g., *STK11* and *PTEN*). For those, we will estimate the relative risks by calculating incidence ratios to assess the differences in incidences between family members carrying a PPV and family members not carrying the variant, and will use a Cox proportional hazards model to estimate the hazard ratio (HR).

To define the tumor spectrum associated with alterations in each TUMOSPEC gene, we assume that the TUMOSPEC families are not selected because of the incidence of cancer at sites other than breast and ovary. Therefore, the incidence of these cancers in the recruited families can be studied by comparing it to that of the general population. The expected number of cancers per 5-year age category will be calculated from the French age-, sex- and period-specific estimated incidences. The standardized incidence ratio (SIR) of cancer associated with PPVs will be estimated from the ratio between the observed and the expected number of cases in the families. We will also calculate the relative risk weighed on the a priori probability of being a PPV carrier [36]. We will correct for bias by the selective testing of survivors and/or relatives affected by cancer among families, if any. Indeed, the over genotyping of cases may bias towards the null hypothesis within the categories of relatives with an unknown genotype [37].

All NGS data are now being centralized at the coordinating centre, and another short-term objective is to propose a consensus bioinformatics pipeline for future analyses of TUMOSPEC data. Indeed, currently, each diagnostics laboratory uses its in-house bioinformatics pipeline built for routine tests, and no standardization of the pipelines regarding basic quality control criteria, the version of the reference genome used for mapping or the annotation tools and databases (and version) was requested to characterize the variants. Moreover, the selection criteria for variants’ eligibility are currently being discussed (MAF, in silico tools used to predict the deleteriousness of the variants). The MAF threshold of 0.05% to select eligible variants was a compromise between avoiding the inclusion of too many innocuous variants and not missing PPVs at conserved positions on the genome based on previous work conducted on some of the TUMOSPEC genes [38,39,40,41,42,43]. Some exceptions have been made for some recurrent variants in *CHEK2* and in *FANCM*, and exceptions for other variants may be made in the future. Similarly, the choice of the prediction tools to assess the deleteriousness of the missense variants may not be optimal (CADD, Align-GVGD), and work is underway to assess the performance of other tools.

## 5. Conclusions

We have demonstrated the feasibility of a streamlined national study approach for achieving a large family sample with genetic, clinical and epidemiological data, representing an important resource for the study of cancer risks and the tumor spectrum associated with PPV in cancer susceptibility genes. The TUMOSPEC feasibility study involved nearly 4500 index cases recruited between September 2017 and December 2019 along with their relatives. This showed that the recruitment processes are well adapted to the clinical and laboratory constraints and that communication between the various partners (clinicians, biologists, coordinating centre and study participants) was smooth. Our planned larger study will amplify this resource and will allow us to gather a sufficient number of positive families for each investigated gene in a reasonable period of time. The final goal of this national effort is to improve the understanding of the cancer risk levels associated with the different types of rare variants for each gene and to provide appropriate clinical management guidelines. The knowledge, know-how and data that will emanate from the TUMOSPEC protocol will pave the way for future studies with extended gene panels or involving populations at a high risk of other cancer types. The same rapid discovery of new susceptibility genes is seen in all fields of cancer genetics, with the same lack of information. Hence, the framework of this protocol may rapidly be adapted for the study of other familial cancers.

## Figures and Tables

**Table 1:** Available data per type of participant.

Dataset	Participant	Signed Consent Form ^1^	Genetic Test			Questionnaire ^3^	
			Negative ^2^	Positive	Pending	Sent ^4^	Completed ^5^
Prospective	Index case	4431	1933	456	2042	417	151
	Relative (all)	306	55	28	223	246	145
	1st degree	138	20	22	96	111	64
	2nd degree	69	16	2	51	60	36
	Cousin	99	19	4	76	75	45
Retrospective	Index case	71	*n/a*	71	*n/a*	71	42
	Relative (all)	80	31	16	33	78	55
	1st degree	34	13	14	7	33	22
	2nd degree	16	7	2	7	15	11
	Cousin	30	11	0	19	30	22

^1^ Returned to the coordinating centre by 31 July 2020. ^2^ For index cases: genetic test is negative if no eligible variant was identified in any of the TUMOSPEC genes; for relatives: genetic test is negative if the variant identified in the index case was not found in the relative. *n/a*: not applicable. ^3^ Questionnaires were sent to index cases with a positive genetic test and to all invited relatives. ^4^ Number of questionnaires provided to participants before 31 July 2020. ^5^ Questionnaires completed and sent back to the coordinating centre by 31 July 2020.

**Table 2:** Distribution of index cases, according to result of the genetic analysis.

Dataset	TUMOSPEC Panel	All ^1^	Women with Cancer			Men with Cancer		
		*N* (%)	*N*	Mean Age at Diagnosis of First Cancer	Range	*N*	Mean Age at Diagnosis of First Cancer	Range
Prospective	All	4431 (100)	3983	47.5	(12–86)	75	60.5	(30–88)
	Negative	1933 (43.6)	1669	47.4	(18–86)	26	61.9	(35–88)
	Negative and *BRCA1/2* neg.	432 (9.7)	340	49.2	(21–76)	5	55.2	(38–72)
	Negative and *BRCA1* pos.	32 (0.7)	24	46.7	(31–67)	0	-	-
	Negative and *BRCA2* pos.	24 (0.5)	20	42.1	(29–67)	0	-	-
	Negative, *BRCA1/2* pending	1445 (32.6)	1285	47.0	(18–86)	21	63.5	(35–88)
	Positive	456 (10.3)	415	46.9	(17–84)	8	54.4	(30–72)
	Positive and *BRCA1/2* neg.	421 (9.5)	384	46.9	(17–84)	8	54.4	(30–72)
	Positive and *BRCA1* pos.	14 (0.3) ^2^	12 ^2^	47.6	(27–66)	0	-	-
	Positive and *BRCA2* pos.	19 (0.4) ^2^	17^2^	47.3	(28–81)	0	-	-
	Positive, *BRCA1/2* pending	3 (0.1)	3	43.0	(25–59)	0		
	Pending result	2042 (46.1)	1899	47.8	(12–85)	41	60.8	(34–81)
Retrospective	All (positive)	71 (100)	67	47.7	(26–86)	2	48.5	(32–65)
	Positive and *BRCA1/2* neg.	70 (98.6)	66	47.8	(26–86)	2	48.5	(32–65)
	Positive and *BRCA1* pos.	0 (0)	0	-	-	0	-	-
	Positive and *BRCA2* pos.	1 (1.4)	1	41.0	*n/a*	0	-	-

^1^ Index cases, affected and unaffected with cancer at enrollment in TUMOSPEC. ^2^ One woman carried an MSH2 variant of uncertain clinical significance, a *BRCA1* pathogenic variant and a *BRCA2* pathogenic variant.

## Data Availability

The data underlying this article will be shared upon reasonable request to the corresponding authors. The data presented in this study are available upon request from the corresponding authors. The data are not publicly available due to privacy and ethical restrictions.

## Supplementary Materials

The following are available online at https://www.mdpi.com/article/10.3390/cancers13153659/s1. Table S1: Molecular diagnostics laboratories involved in the feasibility study. Table S2: Family cancer clinics involved in the feasibility study. Table S3: Variants identified in index cases included during the three-year feasibility study. Figure S1: Dynamic of return rate of questionnaires, according to type of participants. X axis: number of questionnaires. Q-Index sent: questionnaires sent to index cases carrying a PPV; Q-Index received: filled-in questionnaires from index cases returned to the coordinating centre. Q-Relatives sent: questionnaires sent to relatives; Q-Relatives received: filled-in questionnaires from relatives returned to the coordinating centre. Return rates for questionnaires per type of participant are indicated on the graph (%).

## Appendix A

The following clinics and diagnostics laboratories composed the TUMOSPEC Investigators Group:

***Clinics.*** Institut Curie, Paris and Saint Cloud: Dominique Stoppa-Lyonnet; Gustave Roussy, Villejuif: Olivier Caron; Clinique Pasteur, Toulouse: Mathilde Martinez; Institut de Cancérologie Jean Godinot, Reims: Tan Dat Nguyen; Centre Eugène Marquis, Rennes: Louise Crivelli; Centre Paul Strauss and Institut de cancérologie Strasbourg Europe: Hélène Schuster; Institut Bergonié, Bordeaux: Virginie Bubien; Centre Hospitalier Georges Renon: Paul Gesta; Centre Georges François Leclerc, Dijon: Laurence Faivre, Sophie Nambot, Caroline Sawka, Amandine Baurand, Elodie Cosset, Allan Lancon; CHU Hôpital Nord, Saint Etienne: Fabienne Prieur; Centre Antoine Lacassagne, Nice: Véronique Mari; Institut de Cancérologie de l’Ouest—Site Paul Papin, Angers: Marie-Emmanuelle Morin-Meschin; Hôpital Pellegrin-Tripode, Bordeaux: Julie Tinat; Groupe Confluent, Nantes: Alain Lortholary; Centre René Gauducheau, Saint Herblain: Capucine Delnatte; CHU de Grenoble: Hélène Dreyfus; CHRU—Hôpital Jean Minjoz, Besançon: Marie-Agnès Collonge-Rame; Centre Hospitalier de Troyes, Monique Mozelle-Nivoix; Institut Paoli Calmettes, Marseille: Catherine Noguès, Jessica Moretta; Institut Claudius Regaud, Toulouse: Laurence Gladieff; Hospices Civils de Lyon, Bron: Sophie Giraud; Centre hospitalier d’Angoulême, Stéphanie Chieze-Valero; Centre hospitalier de la Rochelle: Lucie Salle; Centre Hospitalier—Hôpital d’enfants, Dijon: Laurence Faivre; Centre Hospitalier La Milétrie, Poitiers: Paul Gesta; Polyclinique Courlancy, Reims: Fanny Brayotel; CHU Rouen Normandie, Rouen: Thierry Frebourg (deceased prematurely); CHU de Nîmes—Hôpital Caremeau, Nîmes: Jean Chiesa, Audrey Lamouroux, Philippe Khau Van Kien; Hôpital Sud, Rennes, Philippe Denizeau; Centre Jean Perrin, Clermont-Ferrand: Yves-Jean Bignon; Hôpital Saint Louis, Paris: Odile Cohen-Haguenauer; Centre Oscar Lambret, Lille: Audrey Mailliez; Institut Sainte Catherine, Avignon: Hélène Dreyfus; Hôpital Arnaud de Villeneuve, Montpellier: Isabelle Coupier; Centre médical de Bligny, Briis-sous-Forges: Olivier Caron; Hôpital Jacques Monod, Le Havre: Elodie Lacaze; CHU Clarac, Fort-de-France: Odile Béra; Centre Léon Bérard, Lyon: Christine Lasset; Hôpitaux Universitaires Pitié Salpêtrière—Charles Foix, Paris: Patrick Benusiglio; Centre de Radiothérapie Hartmann, Levallois-Perret: Pascal Pujol; Centre Hospitalier, Tours: Edouard Cottereau; CHU—Hôpital Nord, Saint Etienne: Fabienne Prieur; Clinique Clémentville, Montpellier: Pascal Pujol; Centre Hospitalier Régional, Orléans: Edouard Cottereau; Centre Hospitalier Jacques Cœur, Bourges: Isabelle Mortemousque; Hôpital Pasteur, Colmar: Jean-Marc Limacher.

***Diagnostics laboratories.*** Institut Curie, Paris: Lisa Golmard; Centre François Baclesse, Caen: Dominique Vaur; Gustave Roussy, Villejuif: Etienne Rouleau, Marine Guillaud-Bataille, Odile Cabaret, Alice Fiévet; CHU de Nantes: Céline Garrec; Institut Bergonié, Bordeaux: Nicolas Sevenet; Institut Claudius Regaud, Toulouse: Christine Toulas; Centre Georges François Leclerc, Dijon: Vincent Goussot; Institut Paoli Calmettes, Marseille: Cornel Popovici; Centre Léon Bérard, Lyon: Nadia Boutry-Kryza; Institut de Cancérologie Jean Godinot, Reims: Fanny Brayotel; CHU de Grenoble: Marie Bidart; Centre Paul Strauss, Strasbourg: Danièle Muller; Centre Jean Perrin, Clermont-Ferrand: Nancy Uhrhammer; Hôpitaux Universitaires Pitié Salpêtrière, Paris: Florence Coulet; Hôpital Saint Louis, Paris: Paul Vilquin; Hôpital Arnaud de Villeneuve, Montpellier: Jean-Marc Rey; Hôpital de Hautepierre, Strasbourg: Christine Maugard.
