# Supplementary material for: TUMOSPEC: A Nation-Wide Study of Hereditary Breast and Ovarian Cancer Families with a Predicted Pathogenic Variant Identified through Multigene Panel Testing
Source: Cancers (Basel). 2021 Jul 21;13(15):3659. doi: 10.3390/cancers13153659 (PMC8345200; doi:10.3390/cancers13153659)
Supplement: Supplementary file 1 [file cancers-13-03659-s001.zip › cancers-1273124-supplementary.pdf]

Table S1. Molecular diagnostics laboratories involved in the feasibility study.

Laboratory name	Institute	City
Service de Génétique	Institut Curie	Paris
Laboratoire de Biologie et de Génétique du Cancer	Centre François Baclesse	Caen
Service de Génétique	Gustave Roussy	Villejuif
Laboratoire de Génétique moléculaire	CHU de Nantes	Nantes
Laboratoire de Génétique moléculaire	Institut Bergonié	Bordeaux
Laboratoire d'Oncogénétique	Institut Claudius Regaud	Toulouse
Département de Biologie et Pathologie des Tumeurs	Centre Georges François Leclerc	Dijon
Laboratoire d'Oncogénétique Moléculaire	Institut Paoli Calmettes	Marseille
Laboratoire de génétique constitutionnelle des cancers fréquents	Centre Léon Bérard	Lyon
Laboratoire d'Oncogénétique	Institut de Cancérologie Jean Godinot	Reims
Laboratoire de génétique moléculaire : maladies rares et oncologies	CHU de Grenoble	Grenoble
Laboratoire d'Oncogénétique	Centre Paul Strauss	Strasbourg
LBM OncoGenAuvergne	Centre Jean Perrin	Clermont-Ferrand
Laboratoire d'Oncogénétique et Angiogénétique Moléculaire	Hôpitaux Universitaires Pitié Salpêtrière	Paris
Département de Génomique des Tumeurs solides	Hôpital Saint Louis	Paris
Laboratoire de Biopathologie cellulaire et tissulaire des tumeurs	Hôpital Arnaud de Villeneuve	Montpellier

Table S2. Family cancer clinics involved in the feasibility study.

Family cancer clinics	City	Reference laboratory
Institut Curie	Paris and Saint Cloud	Institut Curie, Paris
Gustave Roussy	Villejuif	Gustave Roussy, Villejuif
Clinique Pasteur	Toulouse	Institut Claudius Regaud, Toulouse
Institut de Cancérologie Jean Godinot	Reims	Institut Jean Godinot, Reims
Centre Eugène Marquis	Rennes	Centre François Baclesse, Caen
Centre Paul Strauss / Institut de Cancérologie Strasbourg Europe	Strasbourg	Gustave Roussy, Villejuif; Centre Paul Strauss, Strasbourg
Institut Bergonié	Bordeaux	Institut Bergonié, Bordeaux
Centre Hospitalier Georges Renon	Niort	Centre François Baclesse, Caen;<br>CHU Hôtel Dieu, Nantes; Gustave Roussy, Villejuif
Centre Georges François Leclerc	Dijon	Centre Georges François Leclerc, Dijon
Centre Antoine Lacassagne	Nice	Institut Paoli Calmettes, Marseille
CHU – Hôpital Nord	Saint Etienne	Centre Léon Bérard, Lyon
Institut de Cancérologie de l'Ouest - Site Paul Papin	Angers	CHU Hôtel Dieu, Nantes; Centre François Baclesse, Caen
Hôpital Pellegrin-Tripode	Bordeaux	Institut Bergonié, Bordeaux
Centre René Gauducheau	Saint Herblain	CHU Hôtel Dieu, Nantes
Groupe Confluent	Nantes	CHU Hôtel Dieu, Nantes; Institut Curie, Paris
CHU de Grenoble	Grenoble	CHU de Grenoble
CHRU - Hôpital Jean Minjoz	Besançon	Centre Georges François Leclerc, Dijon
Centre Hospitalier de Troyes	Troyes	Institut Curie, Paris
Institut Claudius Regaud	Toulouse	Institut Claudius Regaud, Toulouse
Institut Paoli Calmettes	Marseille	Institut Paoli Calmettes, Marseille
Hospices Civils de Lyon	Bron	Centre Léon Bérard, Lyon

Centre Hospitalier de Girac de Saint Michel	Angoulême	Centre François Baclesse, Caen; CHU Hôtel Dieu, Nantes
Centre Hospitalier de la Rochelle	La Rochelle	Centre François Baclesse, Caen; CHU Hôtel Dieu, Nantes
Centre Hospitalier – Hôpital d'enfants	Dijon	Centre Georges François Leclerc, Dijon
Centre Hospitalier La Milétrie	Poitiers	Centre François Baclesse, Caen; CHU Hôtel Dieu, Nantes
Polyclinique Courlancy	Reims	Institut Curie, Paris
CHU Rouen Normandie	Rouen	Centre François Baclesse, Caen
CHU de Nîmes - Hôpital Caremeau	Nîmes	Institut Curie, Paris
Hôpital Sud	Rennes	Centre François Baclesse, Caen
Centre Jean Perrin	Clermont-Ferrand	Centre Jean Perrin, Clermont-Ferrand
Hôpital Saint Louis	Paris	Hôpital La Pitié - Salpétrière, Paris; Hôpital Saint Louis, Paris
Centre Oscar Lambret	Lille	Centre Oscar Lambret, Lille
Institut Sainte Catherine	Avignon	Institut Paoli Calmettes, Marseille
Hôpital Arnaud de Villeneuve	Montpellier	Centre François Baclesse, Caen
Centre médical de Bligny	Briis-sous-Forges	Gustave Roussy, Villejuif
Hôpital Jacques Monod	Le Havre	Centre François Baclesse, Caen
CHU Clarac	Fort-de-France	Centre François Baclesse, Caen

Table S3. Variants identified in index cases during the feasibility study. LoF, loss-of-function; in-frame indel, in-frame insertion–deletion.

Gene	Transcript	HGVS nomenclature	Effect on protein	Variant type	No. of carriers among prospective index cases	No. of carriers among retrospective index cases
ATM	NM_000051.3	c.68G>A	p.Arg23Gln	Missense	1	0
ATM	NM_000051.3	c.610G>A	p.Gly204Arg	Missense	3	0
ATM	NM_000051.3	c.716T>G	p.Phe239Cys	Missense	1	0
ATM	NM_000051.3	c.748C>T	p.Arg250*	LoF	1	0
ATM	NM_000051.3	c.946T>C	p.Tyr316His	Missense	2	0
ATM	NM_000051.3	c.1009C>T	p.Arg337Cys	Missense	3	1
ATM	NM_000051.3	c.1010G>A	p.Arg337His	Missense	2	0
ATM	NM_000051.3	c.1021G>A	p.Val341Ile	Missense	1	0
ATM	NM_000051.3	c.1066-1G>A	p.?	LoF	1	0
ATM	NM_000051.3	c.1126G>T	p.Glu376*	LoF	1	0
ATM	NM_000051.3	c.1402_1403del	p.Lys468Glufs*18	LoF	2	0
ATM	NM_000051.3	c.1464G>T	p.Trp488Cys	Missense	2	0
ATM	NM_000051.3	c.1477C>T	p.Arg493Cys	Missense	1	0
ATM	NM_000051.3	c.1577T>C	p.Leu526Ser	Missense	1	0
ATM	NM_000051.3	c.1607+1G>A	p.?	LoF	0	1
ATM	NM_000051.3	c.1610C>A	p.Pro537His	Missense	1	0
ATM	NM_000051.3	c.1726A>G	p.Ile576Val	Missense	1	0
ATM	NM_000051.3	c.1898+2T>G	p.?	LoF	0	1
ATM	NM_000051.3	c.2254_2255del	p.Leu752Asnfs*12	LoF	0	1
ATM	NM_000051.3	c.2254C>G	p.Leu752Val	Missense	0	1
ATM	NM_000051.3	c.2450A>T	p.Asp817Val	Missense	1	0
ATM	NM_000051.3	c.2921+1G>T	p.?	LoF	0	1
ATM	NM_000051.3	c.2922-2A>G	p.?	LoF	1	0
ATM	NM_000051.3	c.3149T>G	p.Leu1050Arg	Missense	1	0

ATM	NM_000051.3	c.3256C>T	p.Arg1086Cys	Missense	1	0
ATM	NM_000051.3	c.3279_3282delCAAT	p.Asn1094Aspfs*14	LoF	0	1
ATM	NM_000051.3	c.3629T>C	p.Met1210Thr	Missense	1	0
ATM	NM_000051.3	c.3712_3716del	p.Leu1238Lysfs*6	LoF	3	0
ATM	NM_000051.3	c.3756_3757dupTA	p.Lys1253Ilefs*4	LoF	0	1
ATM	NM_000051.3	c.3802del	p.Val1268*	LoF	3	2
ATM	NM_000051.3	c.3994-1G>C	p.?	LoF	0	1
ATM	NM_000051.3	c.3994-1G>T	p.?	LoF	1	0
ATM	NM_000051.3	c.4324T>C	p.Tyr1442His	Missense	1	0
ATM	NM_000051.3	c.4396C>T	p.Arg1466*	LoF	1	0
ATM	NM_000051.3	c.4414T>G	p.Leu1472Val	Missense	1	0
ATM	NM_000051.3	c.468G>A	p.Trp156*	LoF	0	1
ATM	NM_000051.3	c.4724G>A	p.Arg1575His	Missense	1	0
ATM	NM_000051.3	c.4852C>T	p.Arg1618*	LoF	2	0
ATM	NM_000051.3	c.4853G>A	p.Arg1618Gln	Missense	1	0
ATM	NM_000051.3	c.4909+1G>A	p.?	LoF	1	0
ATM	NM_000051.3	c.4916C>T	p.Pro1639Leu	Missense	2	0
ATM	NM_000051.3	c.5066_5085del	p.Gln1689Leufs*6	LoF	1	0
ATM	NM_000051.3	c.5144T>C	p.Leu1715Pro	Missense	0	1
ATM	NM_000051.3	c.5185G>C	p.Val1729Leu	Missense	1	0
ATM	NM_000051.3	c.5189G>T	p.Arg1730Leu	Missense	1	0
ATM	NM_000051.3	c.5210T>G	p.Leu1737Trp	Missense	1	0
ATM	NM_000051.3	c.5301del	p.Phe1767fs	LoF	1	0
ATM	NM_000051.3	c.5554C>T	p.Gln1852*	LoF	0	2
ATM	NM_000051.3	c.5657C>T	p.Pro1886Leu	Missense	1	0
ATM	NM_000051.3	c.5692C>T	p.Arg1898*	LoF	1	0
ATM	NM_000051.3	c.5706_5707insA	p.Asp1902Glufs*27	LoF	0	1
ATM	NM_000051.3	c.5728C>A	p.Leu1910Ile	Missense	1	0
ATM	NM_000051.3	c.5750G>C	p.Arg1917Thr	Missense	1	0
ATM	NM_000051.3	c.5973A>C	p.Glu1991Asp	Missense	1	0
ATM	NM_000051.3	c.6059G>T	p.Gly2020Val	Missense	1	0
ATM	NM_000051.3	c.6115G>A	p.Glu2039Lys	Missense	0	1
ATM	NM_000051.3	c.6820G>A	p.Ala2274Thr	Missense	1	0

ATM	NM_000051.3	c.6891A>C	p.Gln2297His	Missense	1	0
ATM	NM_000051.3	c.7091C>A	p.Ala2364Glu	Missense	1	0
ATM	NM_000051.3	c.7166C>T	p.Ser2389Leu	Missense	1	0
ATM	NM_000051.3	c.7223C>T	p.Ser2408Leu	Missense	1	0
ATM	NM_000051.3	c.7271T>G	p.Val2424Gly	Missense	0	2
ATM	NM_000051.3	c.7375C>G	p.Arg2459Gly	Missense	1	0
ATM	NM_000051.3	c.7468C>T	p.Leu2490Phe	Missense	1	0
ATM	NM_000051.3	c.7522G>A	p.Gly2508Arg	Missense	1	0
ATM	NM_000051.3	c.7575C>G	p.Arg2459Gly	Missense	1	0
ATM	NM_000051.3	c.7618G>A	p.Val2540Ile	Missense	2	0
ATM	NM_000051.3	c.7658C>A	p.Pro2553His	Missense	1	0
ATM	NM_000051.3	c.7788+1G>C	p.?	LoF	2	0
ATM	NM_000051.3	c.7919C>T	p.Thr2640Ile	Missense	1	0
ATM	NM_000051.3	c.7943del	p.Pro2648Glnfs*12	LoF	1	0
ATM	NM_000051.3	c.8147T>C	p.Val2716Ala	Missense	1	0
ATM	NM_000051.3	c.8191G>C	p.Val2731Leu	Missense	1	0
ATM	NM_000051.3	c.8228C>T	p.Thr2743Met	Missense	1	0
ATM	NM_000051.3	c.8264_8268del	p.Gly2718_Lys2756del39	LoF	1	0
ATM	NM_000051.3	c.8327T>C	p.Ile2776Thr	Missense	1	0
ATM	NM_000051.3	c.8494C>T	p.Arg2832Cys	Missense	0	1
ATM	NM_000051.3	c.8545C>T	p.Arg2849*	LoF	1	0
ATM	NM_000051.3	c.8560C>T	p.Arg2854Cys	Missense	2	0
ATM	NM_000051.3	c.8561G>A	p.Arg2854His	Missense	1	0
ATM	NM_000051.3	c.8614C>A	p.His2872Asn	Missense	1	0
ATM	NM_000051.3	c.8624A>G	p.Asn2875Ser	Missense	1	0
ATM	NM_000051.3	c.8734A>G	p.Arg2912Gly	Missense	1	0
ATM	NM_000051.3	c.8741T>C	p.Ile2914Thr	Missense	1	0
ATM	NM_000051.3	c.8810T>C	p.Val2937Ala	Missense	1	0
ATM	NM_000051.3	c.8860T>C	p.Tyr2954His	Missense	1	0
ATM	NM_000051.3	c.8969A>G	p.Glu2990Gly	Missense	1	0
ATM	NM_000051.3	c.9008A>G	p.Asn3003Ser	Missense	1	0
ATM	NM_000051.3	c.9139C>T	p.Arg3047*	LoF	1	0
ATM	NM_000051.3	c.9166G>T	p.Val3056Leu	Missense	1	0

BAP1	NM_004656.3	c.338G>C	p.Ser113Thr	Missense	1	0
BAP1	NM_004656.3	c.623G>A	p.Arg208Gln	Missense	1	0
BAP1	NM_004656.3	c.637C>T	p.Arg213Cys	Missense	1	0
BAP1	NM_004656.3	c.944A>C	p.Glu315Ala	Missense	0	1
BAP1	NM_004656.3	c.1154G>A	p.Arg385Gln	Missense	1	0
BARD1	NM_000465.3	c.30_44dup	p.Gln11_Arg15dup	In-frame indel	1	0
BARD1	NM_000465.3	c.158G>A	p.Cys53Tyr	Missense	1	0
BARD1	NM_000465.3	c.213T>A	p.Cys71*	LoF	1	0
BARD1	NM_000465.3	c.365-?_1314+?	p.?	LoF	1	0
BARD1	NM_000465.3	c.1339C>G	p.Leu447Val	Missense	2	0
BARD1	NM_000465.3	c.1652C>G	p.Ser551*	LoF	1	0
BARD1	NM_000465.3	c.1690C>T	p.Gln564*	LoF	1	0
BARD1	NM_000465.3	c.1693C>T	p.Arg565Cys	Missense	2	0
BARD1	NM_000465.3	c.1868G>A	p.Gly623Glu	Missense	1	0
BARD1	NM_000465.3	c.1915T>C	p.Cys639Arg	Missense	2	0
BARD1	NM_000465.3	c.1973G>A	p.Arg658His	Missense	1	0
BARD1	NM_000465.3	c.2161G>A	p.Ala721Thr	Missense	1	0
BARD1	NM_000465.3	c.2233T>G	p.Tyr745Asp	Missense	1	0
BRIP1	NM_032043.2	c.128_131del	p.Leu43Trpfs*11	LoF	1	0
BRIP1	NM_032043.2	c.139C>G	p.Pro47Ala	Missense	1	0
BRIP1	NM_032043.2	c.316C>T	p.Arg106Cys	Missense	1	0
BRIP1	NM_032043.2	c.379+1G>A	p.?	LoF	1	0
BRIP1	NM_032043.2	c.415T>G	p.Ser139Ala	Missense	2	0
BRIP1	NM_032043.2	c.484C>T	p.Arg162*	LoF	0	1
BRIP1	NM_032043.2	c.529G>A	p.Gly177Arg	Missense	1	0
BRIP1	NM_032043.2	c.550G>T	p.Asp184Tyr	Missense	1	0
BRIP1	NM_032043.2	c.643del	p.Ser215Leufs*59	LoF	1	0
BRIP1	NM_032043.2	c.838G>T	p.Asp280Tyr	Missense	1	0
BRIP1	NM_032043.2	c.958del	p.Ser320Valfs*18	LoF	1	0
BRIP1	NM_032043.2	c.1066C>T	p.Arg356*	LoF	1	0
BRIP1	NM_032043.2	c.1207C>T	p.Arg403Trp	Missense	1	0
BRIP1	NM_032043.2	c.1372G>T	p.Glu458*	LoF	1	0
BRIP1	NM_032043.2	c.1425_1429delAACTT	p.Leu475Phefs*34	LoF	1	0

BRIP1	NM_032043.2	c.1442G>T	p.Gly481Val	Missense	1	0
BRIP1	NM_032043.2	c.1474_2097dup	p.?	LoF	1	0
BRIP1	NM_032043.2	c.1586G>A	p.Gly529Glu	Missense	1	0
BRIP1	NM_032043.2	c.1641T>G	p.Asp547Glu	Missense	1	0
BRIP1	NM_032043.2	c.1798T>C	p.Phe600Leu	Missense	1	0
BRIP1	NM_032043.2	c.1871C>T	p.Ser624Leu	Missense	1	0
BRIP1	NM_032043.2	c.2047G>C	p.Val683Leu	Missense	1	0
BRIP1	NM_032043.2	c.2087C>T	p.Pro696Leu	Missense	1	0
BRIP1	NM_032043.2	c.2253dupG	p.Lys752Glufs*13	LoF	0	1
BRIP1	NM_032043.2	c.2258_2379del	p.Asp753Glyfs*2	LoF	1	0
BRIP1	NM_032043.2	c.2325T>G	p.Asn775Lys	Missense	1	0
BRIP1	NM_032043.2	c.2392C>T	p.Arg798*	LoF	1	0
BRIP1	NM_032043.2	c.2400C>G	p.Tyr800*	LoF	2	0
BRIP1	NM_032043.2	c.2684_2687delCCAT	p.Ser895*	LoF	1	0
BRIP1	NM_032043.2	c.2768T>C	p.Leu923Pro	Missense	1	0
BRIP1	NM_032043.2	c.2774C>G	p.Ala925Gly	Missense	1	0
BRIP1	NM_032043.2	c.2990_2993del	p.Thr997Argfs*61	LoF	1	0
CDH1	NM_004360.3	c.1-?_48+?del	p.?	LoF	1	0
CDH1	NM_004360.3	c.8C>G	p.Pro3Arg	Missense	1	0
CDH1	NM_004360.3	c.41_46dup	p.Leu14_Leu15dup	In-frame indel	1	0
CDH1	NM_004360.3	c.44_46dup	p.Leu14_Leu15dup	In-frame indel	1	0
CDH1	NM_004360.3	c.214G>A	p.Asp72Asn	Missense	1	0
CDH1	NM_004360.3	c.344C>T	p.Thr115Met	Missense	1	0
CDH1	NM_004360.3	c.854C>T	p.Thr285Ile	Missense	1	0
CDH1	NM_004360.3	c.1003C>T	p.Arg335*	LoF	1	0
CDH1	NM_004360.3	c.1004G>A	p.Arg335Gln	Missense	1	0
CDH1	NM_004360.3	c.1009_1010del	p.Ser337Phefs*12	LoF	1	0
CDH1	NM_004360.3	c.1137G>A	p.Thr379Thr	LoF	1	0
CDH1	NM_004360.3	c.1360G>A	p.Val454Ile	Missense	1	0
CDH1	NM_004360.3	c.1571G>A	p.Arg524Gln	Missense	1	0
CDH1	NM_004360.3	c.1603A>T	p.Ile535Phe	Missense	1	0
CDH1	NM_004360.3	c.2017C>A	p.Gln673Lys	Missense	1	0
CDH1	NM_004360.3	c.2179C>T	p.Leu727Phe	Missense	1	0

CDH1	NM_004360.3	c.2245C>T	p.Arg749Trp	Missense	1	0
CDH1	NM_004360.3	c.2326C>A	p.Leu776Met	Missense	1	0
CDH1	NM_004360.3	c.2336G>A	p.Arg779Gln	Missense	1	0
CDH1	NM_004360.3	c.2343A>T	p.Glu781Asp	Missense	1	0
CDH1	NM_004360.3	c.2398del	p.Arg800Alafs*16	LoF	1	0
CDH1	NM_004360.3	c.2399G>A	p.Arg800His	Missense	2	0
CDH1	NM_004360.3	c.2635G>A	p.Gly879Ser	Missense	2	0
CDH1	NM_004360.3	c.2644G>A	p.Asp882Asn	Missense	1	0
CHEK2	NM_007194.3	c.28C>T	p.Arg361His	Missense	1	0
CHEK2	NM_007194.3	c.123del	p.Ser42Leufs*19	LoF	1	0
CHEK2	NM_007194.3	c.190G>A	p.Arg346Cys	Missense	1	1
CHEK2	NM_007194.3	c.254C>G	p.Asp347Asn	Missense	1	0
CHEK2	NM_007194.3	c.302A>G	p.Ile364Thr	Missense	1	0
CHEK2	NM_007194.3	c.313A>C	p.Thr367Metfs*15	LoF	6	2
CHEK2	NM_007194.3	c.394A>G	p.Met381Val	Missense	1	0
CHEK2	NM_007194.3	c.433C>T	p.Tyr390Ser	Missense	2	0
CHEK2	NM_007194.3	c.434G>A	p.Ala392Val	Missense	1	0
CHEK2	NM_007194.3	c.444+1G>A	p.Pro393Alafs*19	LoF	1	0
CHEK2	NM_007194.3	c.478A>G	p.Tyr404Valfs*2	LoF	1	0
CHEK2	NM_007194.3	c.483_485del	p.Trp411Gly	Missense	2	0
CHEK2	NM_007194.3	c.499G>A	p.Ser422Asn	Missense	2	0
CHEK2	NM_007194.3	c.590A>T	p.Glu457fs	LoF	1	0
CHEK2	NM_007194.3	c.591del	p.Ser465Asn	Missense	1	0
CHEK2	NM_007194.3	c.650G>T	p.Thr476Met	Missense	1	0
CHEK2	NM_007194.3	c.685A>C	p.Leu498Valfs*2	LoF	1	0
CHEK2	NM_007194.3	c.688G>T	p.Arg519Leu	Missense	2	1
CHEK2	NM_007194.3	c.715G>A	p.Thr533Glnfs*33	LoF	0	1
CHEK2	NM_007194.3	c.720del	p.Thr576Glnfs*33	LoF	1	0
CHEK2	NM_007194.3	c.767C>T	p.Glu64Lys	Missense	1	2
CHEK2	NM_007194.3	c.817_818del	p.Pro85Arg	Missense	1	0
CHEK2	NM_007194.3	c.846+1G>C	p.Gln10*	LoF	1	0
CHEK2	NM_007194.3	c.856T>C	p.Asp101Gly	Missense	2	0
CHEK2	NM_007194.3	c.876dupT	p.Asn105His	Missense	1	0

CHEK2	NM_007194.3	c.880A>T	p.Arg117Gly	Missense	0	1
CHEK2	NM_007194.3	c.1036C>T	p.Arg145Trp	Missense	2	0
CHEK2	NM_007194.3	c.1039G>A	p.Arg145Gln	Missense	1	0
CHEK2	NM_007194.3	c.1082G>A	p.?	LoF	1	1
CHEK2	NM_007194.3	c.1091T>C	p.Arg160Gly	Missense	1	0
CHEK2	NM_007194.3	c.1100delC	p.Glu161del	In-frame indel	1	0
CHEK2	NM_007194.3	c.1141A>G	p.Gly167Arg	Missense	1	0
CHEK2	NM_007194.3	c.1169A>C	p.Asn197Ile	Missense	1	0
CHEK2	NM_007194.3	c.1175C>T	p.Val198Phefs*7	LoF	3	0
CHEK2	NM_007194.3	c.1175dup (+/+)	p.Arg217Ile	Missense	1	0
CHEK2	NM_007194.3	c.1209_1233del	p.Asn229His	Missense	1	0
CHEK2	NM_007194.3	c.1231T>G	p.Ala230Ser	Missense	1	0
CHEK2	NM_007194.3	c.1265G>A	p.Glu239Lys	Missense	2	0
CHEK2	NM_007194.3	c.1368dup	p.Val241Phefs*7	LoF	1	0
CHEK2	NM_007194.3	c.1394G>A	p.Pro256Leu	Missense	2	0
CHEK2	NM_007194.3	c.1427C>T	p.Glu273Asnfs*16	LoF	1	0
CHEK2	NM_007194.3	c.1492_1496del	p.?	LoF	1	0
CHEK2	NM_007194.3	c.1556G>T	p.Cys286Arg	Missense	1	0
CHEK2	NM_007194.3	c.1596del	p.Asp293*	LoF	0	1
CHEK2	NM_007194.3	c.1725del	p.Ile294Phe	Missense	1	0
FAM175A	NM_139076.2	c.122C>T	p.Ala41Val	Missense	1	0
FAM175A	NM_139076.2	c.659C>T	p.Ala220Val	Missense	1	0
FAM175A	NM_139076.2	c.826_828del	p.Glu276del	In-frame indel	2	0
FANCM	NM_020937.3	c.2586_2589del	p.Lys863Ilefs*12	LoF	1	0
FANCM	NM_020937.3	c.3259A>T	p.Lys1087*	LoF	1	0
MLH1	NM_000249.3	c.41C>T	p.Thr14Ile	Missense	1	0
MLH1	NM_000249.3	c.102G>C	p.Glu34Asp	Missense	1	0
MLH1	NM_000249.3	c.170A>C	p.Lys57Thr	Missense	1	0
MLH1	NM_000249.3	c.125C>T	p.Ala42Val	Missense	1	0
MLH1	NM_000249.3	c.191A>G	p.Asn64Ser	Missense	1	0
MLH1	NM_000249.3	c.287C>G	p.Thr96Ser	Missense	1	0
MLH1	NM_000249.3	c.376T>A	p.Tyr126Asn	Missense	1	0
MLH1	NM_000249.3	c.454G>A	p.Val152Met	Missense	1	0

MLH1	NM_000249.3	c.522dup	p.Lys175Glufs*17	LoF	1	0
MLH1	NM_000249.3	c.539T>G	p.Val180Gly	Missense	1	0
MLH1	NM_000249.3	c.682C>A	p.Leu228Met	Missense	1	0
MLH1	NM_000249.3	c.928A>G	p.Thr310Ala	Missense	1	0
MLH1	NM_000249.3	c.939A>C	p.Glu313Asp	Missense	1	0
MLH1	NM_000249.3	c.1453G>T	p.Asp485Tyr	Missense	1	0
MLH1	NM_000249.3	c.1490G>A	p.Arg497Gln	Missense	1	0
MLH1	NM_000249.3	c.1820T>A	p.Leu607His	Missense	1	0
MLH1	NM_000249.3	c.1855G>C	p.Ala619Pro	Missense	1	0
MRE11A	NM_005591.3	c.18_19delinsTG	p.Leu7Val	Missense	1	0
MRE11A	NM_005591.3	c.260G>A	p.Arg87Gln	Missense	1	0
MRE11A	NM_005591.3	c.274G>A	p.Glu92Lys	Missense	0	1
MRE11A	NM_005591.3	c.391G>A	p.Asp131Asn	Missense	2	0
MRE11A	NM_005591.3	c.394C>T	p.Pro132Ser	Missense	1	0
MRE11A	NM_005591.3	c.482A>G	p.Lys161Arg	Missense	1	0
MRE11A	NM_005591.3	c.497C>T	p.Pro166Leu	Missense	1	0
MRE11A	NM_005591.3	c.539G>C	p.Gly180Ala	Missense	1	0
MRE11A	NM_005591.3	c.784T>A	p.Tyr262Asn	Missense	1	0
MRE11A	NM_005591.3	c.818C>G	p.Ser273Cys	Missense	1	0
MRE11A	NM_005591.3	c.1139G>A	p.Arg380His	Missense	2	0
MRE11A	NM_005591.3	c.1463G>A	p.Arg488His	Missense	1	0
MRE11A	NM_005591.3	c.1883G>A	p.Arg628Lys	Missense	1	0
MRE11A	NM_005591.3	c.1960_1979dup	p.Lys661Thrfs*45	LoF	1	0
MRE11A	NM_005591.3	c.2083_2085del	p.Asp695del	In-frame indel	1	0
MRE11A	NM_005591.3	c.2083_2085dup	p.Asp695dup	In-frame indel	1	0
MSH2	NM_000251.2	c.7G>A	p.Val3Met	Missense	1	0
MSH2	NM_000251.2	c.47A>C	p.Glu16Ala	Missense	1	0
MSH2	NM_000251.2	c.112G>C	p.Asp38His	Missense	1	0
MSH2	NM_000251.2	c.128A>G	p.Tyr43Cys	Missense	1	0
MSH2	NM_000251.2	c.138C>G	p.His46Gln	Missense	3	0
MSH2	NM_000251.2	c.166G>A	p.Glu56Lys	Missense	1	0
MSH2	NM_000251.2	c.482T>C	p.Val161Ala	Missense	1	0
MSH2	NM_000251.2	c.557A>G	p.Asn186Ser	Missense	0	1

MSH2	NM_000251.2	c.562G>C	p.Glu188Gln	Missense	0	1
MSH2	NM_000251.2	c.728G>A	p.Arg243Gln	Missense	2	0
MSH2	NM_000251.2	c.815C>T	p.Ala272Val	Missense	1	0
MSH2	NM_000251.2	c.1087G>T	p.Val363Leu	Missense	1	0
MSH2	NM_000251.2	c.1179G>T	p.Gln593His	Missense	1	0
MSH2	NM_000251.2	c.1413A>C	p.Lys47Asn	Missense	1	0
MSH2	NM_000251.2	c.1472A>C	p.Lys491Thr	Missense	1	0
MSH2	NM_000251.2	c.1601G>T	p.Arg534Leu	Missense	0	1
MSH2	NM_000251.2	c.1915C>A	p.His639Asn	Missense	1	0
MSH2	NM_000251.2	c.2164G>A	p.Val722Ile	Missense	0	1
MSH2	NM_000251.2	c.2377C>A	p.Gln793Lys	Missense	1	0
MSH2	NM_000251.2	c.2422G>T	p.Glu808*	LoF	1	0
MSH2	NM_000251.2	c.2439G>A	p.Met813Ile	Missense	1	0
MSH2	NM_000251.2	c.2500G>A	p.Ala834Thr	Missense	1	0
MSH2	NM_000251.2	c.2533A>G	p.Lys845Glu	Missense	1	0
MSH6	NM_000179.2	c.275_276delinsTG	p.Pro92Leu	Missense	1	0
MSH6	NM_000179.2	c.470A>T	p.Lys157Met	Missense	0	1
MSH6	NM_000179.2	c.817G>A	p.Gly273Arg	Missense	2	0
MSH6	NM_000179.2	c.884A>G	p.Lys295Arg	Missense	2	0
MSH6	NM_000179.2	c.1019T>C	p.Phe340Ser	Missense	1	0
MSH6	NM_000179.2	c.1120_1122delAAG	p.Lys374del	In-frame indel	1	0
MSH6	NM_000179.2	c.1192G>A	p.Val398Met	Missense	1	0
MSH6	NM_000179.2	c.1223C>G	p.Pro408Arg	Missense	1	0
MSH6	NM_000179.2	c.1390A>T	p.Ile464Phe	Missense	1	0
MSH6	NM_000179.2	c.1569_1572del	p.Tyr524fs	LoF	1	0
MSH6	NM_000179.2	c.1885G>A	p.Asp629Asn	Missense	1	0
MSH6	NM_000179.2	c.1894A>G	p.Lys632Glu	Missense	1	0
MSH6	NM_000179.2	c.1993G>A	p.Glu665Lys	Missense	1	0
MSH6	NM_000179.2	c.2225A>G	p.Asn742Ser	Missense	1	0
MSH6	NM_000179.2	c.2245G>A	p.Gly749Arg	Missense	1	0
MSH6	NM_000179.2	c.2249C>A	p.Thr750Lys	Missense	1	0
MSH6	NM_000179.2	c.2392C>G	p.Leu798Val	Missense	1	0
MSH6	NM_000179.2	c.2419G>A	p.Glu807Lys	Missense	1	0

MSH6	NM_000179.2	c.2533T>C	p.Tyr845His	Missense	1	0
MSH6	NM_000179.2	c.2597A>G	p.Lys866Arg	Missense	1	0
MSH6	NM_000179.2	c.2640_2641insAAA	p.Asp880_Gly881insLys	In-frame indel	1	0
MSH6	NM_000179.2	c.2701C>T	p.Arg901Cys	Missense	1	0
MSH6	NM_000179.2	c.2830A>G	p.Ile944Val	Missense	2	0
MSH6	NM_000179.2	c.3040_3042del	p.Lys1014del	In-frame indel	1	0
MSH6	NM_000179.2	c.3131A>C	p.Tyr1044Ser	Missense	0	1
MSH6	NM_000179.2	c.3260C>G	p.Pro1087Arg	Missense	1	0
MSH6	NM_000179.2	c.3313G>A	p.Gly1105Arg	Missense	1	0
MSH6	NM_000179.2	c.3328C>A	p.Pro1110Thr	Missense	1	0
MSH6	NM_000179.2	c.3394G>A	p.Val1132Ile	Missense	1	0
MSH6	NM_000179.2	c.3411G>C	p.Met1137Ile	Missense	1	0
MSH6	NM_000179.2	c.3477C>A	p.Tyr1159*	LoF	1	0
MSH6	NM_000179.2	c.3565A>G	p.Thr1189Ala	Missense	1	0
MSH6	NM_000179.2	c.3584G>C	p.Ser1195Thr	Missense	1	0
MSH6	NM_000179.2	c.3810G>T	p.Met1270Ile	Missense	1	0
MSH6	NM_000179.2	c.3836G>A	p.Ser1279Asn	Missense	1	0
MSH6	NM_000179.2	c.3961A>G	p.Arg1321Gly	Missense	1	0
MSH6	NM_000179.2	c.3968T>C	p.Phe1323Ser	Missense	0	1
NBN	NM_002485.4	c.156_157delTT	p.Ser53Cysfs*9	LoF	0	1
NBN	NM_002485.4	c.335C>T	p.Pro112Leu	Missense	1	0
NBN	NM_002485.4	c.340G>A	p.Val114Ile	Missense	1	0
NBN	NM_002485.4	c.456G>A	p.Met125Ile	Missense	5	0
NBN	NM_002485.4	c.644G>A	p.Arg215Gln	Missense	1	0
NBN	NM_002485.4	c.657_661delACAAA	p.Lys219Asnfs*16	LoF	0	1
NBN	NM_002485.4	c.706A>G	p.Lys236Glu	Missense	1	0
NBN	NM_002485.4	c.970G>A	p.Asp324Asn	Missense	1	0
NBN	NM_002485.4	c.1098C>G	p.Asp366Glu	Missense	1	0
NBN	NM_002485.4	c.1142del	p.Pro381Glnfs*23	LoF	3	1
NBN	NM_002485.4	c.1273A>T	p.Arg425*	LoF	1	0
NBN	NM_002485.4	c.1987G>T	p.Val663Leu	Missense	1	0
NBN	NM_002485.4	c.2090G>A	p.Gly697Glu	Missense	1	0
NBN	NM_002485.4	c.2140C>T	p.Arg714*	LoF	0	1

PALB2	NM_024675.3	c.11C>T	p.Pro4Leu	Missense	1	0
PALB2	NM_024675.3	c.72delG	p.Arg26Glyfs*7	LoF	0	1
PALB2	NM_024675.3	c.94C>G	p.Leu32Val	Missense	1	0
PALB2	NM_024675.3	c.109C>A	p.Arg37Ser	Missense	1	0
PALB2	NM_024675.3	c.172_175del	p.Gln60Argfs*7	LoF	0	1
PALB2	NM_024675.3	c.249del	p.His83Glnfs*94	LoF	1	0
PALB2	NM_024675.3	c.509_510del	p.Arg170Ilefs*14	LoF	1	0
PALB2	NM_024675.3	c.664_666delTTA	p.Leu222del	In-frame indel	1	0
PALB2	NM_024675.3	c.757_758delCT	p.Leu253Ilefs	LoF	1	0
PALB2	NM_024675.3	c.758dup	p.Ser254Ilefs*3	LoF	1	0
PALB2	NM_024675.3	c.889_892delACTG	p.Thr297fs	LoF	1	0
PALB2	NM_024675.3	c.1037_1041del	p.Lys346Thrfs*13	LoF	1	0
PALB2	NM_024675.3	c.1135A>T	p.Lys379*	LoF	1	0
PALB2	NM_024675.3	c.1192del	p.Val398Cysfs*26	LoF	1	0
PALB2	NM_024675.3	c.1240C>T	p.Arg414*	LoF	1	0
PALB2	NM_024675.3	c.1250C>A	p.Ser417Tyr	Missense	2	0
PALB2	NM_024675.3	c.1424delC	p.Ser475*	LoF	1	0
PALB2	NM_024675.3	c.1435C>A	p.Gln479Lys	Missense	1	0
PALB2	NM_024675.3	c.1543A>T	p.Lys515*	LoF	0	1
PALB2	NM_024675.3	c.1846G>C	p.Asp616His	Missense	1	0
PALB2	NM_024675.3	c.1882_1890del	p.Lys628_Cys630del	In-frame indel	1	1
PALB2	NM_024675.3	c.1883A>C	p.Lys628Thr	Missense	1	0
PALB2	NM_024675.3	c.1915G>T	p.Glu639*	LoF	1	0
PALB2	NM_024675.3	c.1965dupT	p.Pro656Serfs*7	LoF	1	0
PALB2	NM_024675.3	c.1972G>T	p.Glu658*	LoF	1	0
PALB2	NM_024675.3	c.1975_1976del	p.Leu659Glufs*3	LoF	1	0
PALB2	NM_024675.3	c.2257C>T	p.Arg753*	LoF	2	0
PALB2	NM_024675.3	c.2727_2728del	p.Thr911Leufs*16	LoF	1	0
PALB2	NM_024675.3	c.2737C>T	p.His913Tyr	Missense	1	0
PALB2	NM_024675.3	c.2750T>C	p.Val917Ala	Missense	1	0
PALB2	NM_024675.3	c.2835-1G>C	p.?	LoF	1	0
PALB2	NM_024675.3	c.2903C>G	p.Ala968Gly	Missense	1	0
PALB2	NM_024675.3	c.2962C>T	p.Gln988*	LoF	1	0

PALB2	NM_024675.3	c.2964del	p.Val989*	LoF	1	0
PALB2	NM_024675.3	c.3026_3027insA	p.Glu1010*	LoF	1	0
PALB2	NM_024675.3	c.?_-201_3113+1_3114-1del	p.?	LoF	1	0
PALB2	NM_024675.3	c.3114-?_3201+?del	p.Asn1039Glyfs*7	LoF	0	2
PALB2	NM_024675.3	c.(3201+1_3202-1)_(3350+1_3351-1)del	p.Gly1068Valfs*5	LoF	1	0
PALB2	NM_024675.3	c.3247G>A	p.Glu1083Lys	Missense	1	0
PALB2	NM_024675.3	c.3256C>T	p.Arg1086*	LoF	1	0
PALB2	NM_024675.3	c.3296C>T	p.Thr1099Met	Missense	1	0
PALB2	NM_024675.3	c.3320T>C	p.Leu1107Pro	Missense	1	0
PALB2	NM_024675.3	c.3418T>G	p.Trp1140Gly	Missense	1	0
PALB2	NM_024675.3	c.3428T>A	p.Leu1143His	Missense	2	0
PALB2	NM_024675.3	c.3492G>T	p.Trp1164Cys	Missense	0	1
PALB2	NM_024675.3	c.3539T>C	p.Ile1180Thr	Missense	2	0
PALB2	NM_024675.3	c.3554A>G	p.Tyr1185Cys	Missense	1	0
PMS2	NM_000535.5	c.106A>C	p.Ser36Arg	Missense	1	0
PMS2	NM_000535.5	c.137G>T	p.Ser46Ile	Missense	6	0
PMS2	NM_000535.5	c.400C>T	p.Arg134*	LoF	0	1
PMS2	NM_000535.5	c.596G>C	p.Arg199Pro	Missense	1	0
PMS2	NM_000535.5	c.632G>A	p.Arg211Gln	Missense	1	0
PMS2	NM_000535.5	c.823C>T	p.Gln275*	LoF	0	1
PMS2	NM_000535.5	c.833A>G	p.His278Arg	Missense	1	0
PMS2	NM_000535.5	c.857A>G	p.Asp286Gly	Missense	1	0
PMS2	NM_000535.5	c.903G>T	p.Tyr268*	LoF	1	0
PMS2	NM_000535.5	c.964G>A	p.Val322Ile	Missense	1	0
PMS2	NM_000535.5	c.1004A>G	p.Asn335Ser	Missense	5	0
PMS2	NM_000535.5	c.1144+2T>G	p.?	LoF	1	0
PMS2	NM_000535.5	c.1927C>T	p.Gln643*	LoF	1	0
PMS2	NM_000535.5	c.1937G>T	p.Arg646Met	Missense	2	0
PMS2	NM_000535.5	c.2444C>T	p.Ser815Leu	Missense	0	1
PMS2	NM_000535.5	c.2452A>G	p.Ile818Val	Missense	1	0
PMS2	NM_000535.5	c.2533C>G	p.His845Asp	Missense	1	0
PTEN	NM_000314.4	c.377C>A	p.Ala126Asp	Missense	1	0

PTEN	NM_000314.4	c.464A>G	p.Tyr155Cys	Missense	1	0
PTEN	NM_000314.4	c.697C>T	p.Arg233*	LoF	1	0
PTEN	NM_000314.4	c.901G>A	p.Asp301Asn	Missense	1	0
RAD50	NM_005732.3	c.223G>T	p.Glu75*	LoF	1	0
RAD50	NM_005732.3	c.577C>T	p.Arg193Trp	Missense	1	0
RAD50	NM_005732.3	c.673G>A	p.Asp225Asn	Missense	2	0
RAD50	NM_005732.3	c.756+2T>C	p.?	LoF	1	0
RAD50	NM_005732.3	c.761G>A	p.Arg254His	Missense	1	0
RAD50	NM_005732.3	c.785T>G	p.Leu262Arg	Missense	2	0
RAD50	NM_005732.3	c.989A>C	p.Glu330Ala	Missense	1	0
RAD50	NM_005732.3	c.1205A>G	p.Glu402Gly	Missense	1	0
RAD50	NM_005732.3	c.1253_1254del	p.Phe418Cysfs*13	LoF	1	0
RAD50	NM_005732.3	c.1277A>G	p.Gln426Arg	Missense	1	0
RAD50	NM_005732.3	c.1336A>G	p.Lys446Glu	Missense	1	0
RAD50	NM_005732.3	c.1436_1437del	p.Gln479Argfs*6	LoF	1	0
RAD50	NM_005732.3	c.2116C>T	p.Arg706*	LoF	0	1
RAD50	NM_005732.3	c.2165dupA	p.Glu723Glyfs*5	LoF	0	1
RAD50	NM_005732.3	c.2177G>A	p.Arg726His	Missense	1	0
RAD50	NM_005732.3	c.2498_2499del	p.Gln833Argfs*11	LoF	0	1
RAD50	NM_005732.3	c.2929-1G>A	p.?	LoF	1	0
RAD50	NM_005732.3	c.3229C>T	p.Arg1077*	LoF	2	0
RAD50	NM_005732.3	c.3230G>A	p.Arg1077Gln	Missense	1	0
RAD51B	NM_133509.3	c.289G>C	p.Gly97Arg	Missense	1	0
RAD51B	NM_133509.3	c.436G>A	p.Ala146Thr	Missense	1	0
RAD51C	NM_058216.1	c.250A>T	p.Lys84*	LoF	0	1
RAD51C	NM_058216.1	c.404G>A	p.Cys135Tyr	Missense	0	1
RAD51C	NM_058216.1	c.404G>C	p.Cys135Ser	Missense	1	0
RAD51C	NM_058216.1	c.414G>C	p.Leu138Phe	Missense	1	1
RAD51C	NM_058216.1	c.428A>G	p.Gln143Arg	Missense	3	0
RAD51C	NM_058216.1	c.431T>C	p.Ile144Thr	Missense	1	0
RAD51C	NM_058216.1	c.556A>T	p.Lys186*	LoF	0	1
RAD51C	NM_058216.1	c.-102-(571+1_572-1)del	p.?	LoF	0	1
RAD51C	NM_058216.1	c.571+2T>C	p.?	LoF	1	0

RAD51C	NM_058216.1	c.577C>T	p.Arg193*	LoF	1	2
RAD51C	NM_058216.1	c.622_623del	p.Ile208Leufs*7	LoF	0	1
RAD51C	NM_058216.1	c.656T>C	p.Leu219Ser	Missense	1	0
RAD51C	NM_058216.1	c.705+1G>A	p.?	LoF	2	0
RAD51C	NM_058216.1	c.(706-?_1131+?)	p.?	LoF	1	0
RAD51C	NM_058216.1	c.709C>T	p.Arg237*	LoF	0	1
RAD51C	NM_058216.1	c.710G>C	p.Arg237Pro	Missense	1	0
RAD51C	NM_058216.1	c.732delT	p.Ile244Metfs*9	LoF	1	0
RAD51C	NM_058216.1	c.773G>A	p.Arg258His	Missense	1	0
RAD51C	NM_058216.1	c.784T>G	p.Leu262Val	Missense	2	0
RAD51C	NM_058216.1	c.890_899del	p.Leu297Hisfs*2	LoF	0	1
RAD51C	NM_058216.1	c.955C>T	p.Arg319*	LoF	1	0
RAD51C	NM_058216.1	c.965+5G>A	p.Glu303Trpfs*41	LoF	1	0
RAD51C	NM_058216.1	c.1026+5_1026+7delGTA	p.Arg322Serfs*22	LoF	1	3
RAD51D	NM_001142571.1	c.1A>T	p.Met1?	LoF	0	1
RAD51D	NM_001142571.1	c.121C>T	p.Gln41*	LoF	0	1
RAD51D	NM_001142571.1	c.129del	p.Cys43Trpfs*37	LoF	0	1
RAD51D	NM_001142571.1	c.131G>A	p.Gly44Asp	Missense	1	0
RAD51D	NM_001142571.1	c.137C>G	p.Ser46Cys	Missense	1	0
RAD51D	NM_001142571.1	c.140A>G	p.Tyr47Cys	Missense	1	0
RAD51D	NM_001142571.1	c.185C>T	p.Ser62Leu	Missense	1	0
RAD51D	NM_001142571.1	c.491T>C	p.Leu164Pro	Missense	1	0
RAD51D	NM_001142571.1	c.529C>T	p.Gln177*	LoF	0	1
RAD51D	NM_001142571.1	c.556C>T	p.Arg186*	LoF	1	0
RAD51D	NM_002878.3	c.617A>G	p.Asp206Gly	Missense	1	0
RAD51D	NM_001142571.1	c.620C>A	p.Ser207*	LoF	1	0
RAD51D	NM_001142571.1	c.694C>T	p.Arg232*	LoF	3	2
RAD51D	NM_001142571.1	c.715C>T	p.Arg239Trp	Missense	2	0
RAD51D	NM_001142571.1	c.793G>A	p.Gly265Arg	Missense	1	1
RAD51D	NM_001142571.1	c.803G>A	p.Trp268*	LoF	1	0
RAD51D	NM_001142571.1	c.806G>A	p.Ser269Asn	Missense	0	1
RAD51D	NM_001142571.1	c.823C>T	p.Arg275Trp	Missense	1	1
RAD51D	NM_001142571.1	c.898C>T	p.Arg300*	LoF	0	2

RAD51D	NM_001142571.1	c.911G>A	p.Gly304Asp	Missense	1	0
RINT1	NM_021930.5	c.854C>T	p.Thr285Ile	Missense	1	0
RINT1	NM_021930.5	c.943A>G	p.Lys315Glu	Missense	1	0
RINT1	NM_021930.5	c.1949C>T	p.Pro650Leu	Missense	1	0
STK11	NM_000455.4	c.1032_1058del	p.His345_Leu353del	In-frame indel	1	0
STK11	NM_000455.4	c.1150C>T	p.Arg384Trp	Missense	1	0
STK11	NM_000455.4	c.1244G>A	p.Arg415His	Missense	1	0
STK11	NM_000455.4	c.1274G>A	p.Arg425His	Missense	2	0
STK11	NM_000455.4	c.1276C>T	p.Arg426Trp	Missense	1	0
TP53	NM_000546.4	c.31G>A	p.Glu11Lys	Missense	1	0
TP53	NM_000546.4	c.329G>A	p.Arg110His	Missense	1	0
TP53	NM_000546.4	c.379T>C	p.Ser127Pro	Missense	1	0
TP53	NM_000546.4	c.707A>G	p.Tyr236Cys	Missense	1	0
TP53	NM_000546.4	c.847C>T	p.Arg283Cys	Missense	1	0
TP53	NM_000546.4	c.1015G>C	p.Glu339Gln	Missense	1	0
XRCC2	NM_005431.1	c.39+2T>A	p.?	LoF	1	0
XRCC2	NM_005431.1	c.65G>A	p.Ser22Asn	Missense	1	0
XRCC2	NM_005431.1	c.314A>G	p.Glu105Gly	Missense	1	0
XRCC2	NM_005431.1	c.450C>G	p.Ser150Arg	Missense	1	0
XRCC2	NM_005431.1	c.561T>A	p.Tyr187*	LoF	2	0
